# Effects of wetting events on mass timber surface microbial communities and VOC emissions: implications for building operation and occupant well-being

**DOI:** 10.3389/frmbi.2025.1395519

**Published:** 2025-04-09

**Authors:** Gwynne Á. Mhuireach, Susan Collins, Leslie Dietz, Patrick Finn Horve, Aurélie Laguerre, Dale Northcutt, Jason Stenson, Kevin Van Den Wymelenberg, Elliott Gall, Mark Fretz

**Affiliations:** ^1^ Institute for Health in the Built Environment, University of Oregon, Eugene, OR, United States; ^2^ Institute of Molecular Biology, University of Oregon, Eugene, OR, United States; ^3^ Salk Institute for Biological Studies, San Diego, CA, United States; ^4^ Healthy Buildings Research Laboratory, Portland State University, Portland, OR, United States

**Keywords:** shotgun metagenomics, cross-laminated timber, green buildings, terpenes, evidence based design

## Abstract

**Introduction:**

Humans have used wood as a construction material throughout history. Currently, mass timber products, such as cross-laminated timber (CLT), are becoming more popular as a structural material, since they are renewable and have a lower carbon footprint than concrete or steel. Nonetheless, some building types, such as healthcare, veterinary, and food manufacturing, avoid using structural mass timber due to concerns about microbial growth in the event of wetting. One solution is to use protective coatings on mass timber products to increase moisture resistance, although the coatings themselves may generate concerns about volatile organic compound (VOC) emissions. Natural uncoated wood also produces VOCs, some of which may have intrinsic antimicrobial effects.

**Methods:**

In this study, we inoculated coated and uncoated cross- laminated timber (CLT) blocks with a mock microbial community and isolated each block within individual sealed microcosms. We characterized VOCs and surface microbial communities from the CLT blocks before, during, and after wetting periods of varying durations. VOC concentration and emission rate were analyzed with chromatography-mass spectrometry (GC-MS), while microbial community abundance, diversity, and composition were analyzed through qPCR and shotgun metagenomics.

**Results:**

VOC emissions were elevated immediately after inoculation, then decreased through the remainder of the experiment, except for a plateau during the wetting period. VOCs from uncoated CLT blocks were primarily terpenes, while coated blocks emitted VOCs associated with coatings, plastics, and industrial solvents, as well as terpenes. One VOC—acetoin (3-hydroxy, 2-butanone)—was present at high levels across all samples immediately after microbial inoculation. Bacteria comprised 99.54% of the identified microbial sequences. The plastic control microcosm (not containing a CLT block) had higher abundance of viable bacteria for the majority of the study, but there was no difference in abundance between coated and uncoated blocks. Prior to wetting periods, microbial composition was driven primarily by sampling day, whereas surface type played a larger role during and after wetting periods.

## Highlights

- VOC emissions decreased over time.
- Coated CLT generally emitted more VOCs than uncoated.
- More microbial biomass was recovered from the plastic control box than CLT blocks.
- Microbial composition responded differently to wetting, depending on whether blocks were coated or uncoated.

## Introduction

Structural mass timber products, such as cross-laminated timber (CLT), offer a sustainable alternative to conventional construction materials, like concrete and steel (Abed et al., 2022; Comnick et al., 2021; Duan et al., 2022; Puettmann et al., 2021; Skullestad et al., 2016; Tupenaite et al., 2023). In fact, substituting wood for more carbon-intensive materials in half of new urban construction globally could help meet 2030 emissions goals by contributing up to 9% of the needed emissions reduction (Himes and Busby, 2020).

In addition to its environmental benefits, mass timber construction may offer occupant health and comfort benefits (Augustin and Fell, 2015; Burnard and Kutnar, 2015; Fell, 2010; Kotradyova et al., 2019; Nyrud et al., 2010; Sakuragawa et al., 2005; Zhang et al., 2017). Exposed wood in buildings is visually appealing, buffers humidity, and provides a pleasant odor. Studies have shown that occupants experiencing wood environments had more positive emotions, less fatigue, and were more comfortable than those experiencing non-wood environments (Zhang et al., 2016).

Despite its recognized benefits, concern about exposed wood surfaces, including mass timber, persists due to its potential to harbor microorganisms and difficulty of sanitation. However, a large body of research indicates that wood surfaces are generally very low risk for transferring microorganisms compared with other common building and furnishing materials. For instance, Mhuireach et al. (2021) found that viable bacterial load on CLT was lower than concrete, earthen plaster, or painted gypsum board. Numerous other studies in healthcare, food manufacturing, and laboratory environments have concluded that, from a hygiene perspective, wood performs as well, or better, than plastic, vinyl, or steel surfaces (Aviat et al., 2016; Boersig and Cliver, 2010; Koch et al., 2002; Munir et al., 2019). Because wood is porous and microorganisms can enter the fabric of the wood, they become unrecoverable by surface contact after a short period of time (Abrishami et al., 1994; Ak et al., 1994). While surface porosity may partly explain these results, wood also emits a class of volatile organic compounds (VOCs) known as terpenes. These terpenes may contribute to the low survival rates of several pathogens, including methicillin-resistant *Staphylococcus aureus* (MRSA) and *Escherichia coli* (Coughenour, 2009; Da Costa et al., 2008; Greatorex et al., 2011; Pailhoriés et al., 2017; Vainio-Kaila et al., 2017). Although exposure to some VOCs, like benzene and toluene, causes detrimental health effects in humans, terpenes are associated with various health benefits, including reductions in stress, cortisol levels, and heart rate, increase in activity of natural killer cells (lymphocytes that play a key role in the innate immune system), and acting as a natural antimicrobial (Antonelli et al., 2019; Cho et al., 2017; Ikei et al., 2017; Matsubara and Kawai, 2014). Even very high levels of terpenes, such as *α*-pinene and *δ* ^3^-carene, have been demonstrated not to have toxic effects (Gminski et al., 2010; Junge et al., 2021). For softwoods, including pine and fir, which are commonly used in the production of CLT, the majority (70–90%) of VOC emissions are terpenes (Pohleven et al., 2019). On the other hand, it is worth noting that terpenes can also contribute to generation of secondary products, which may be harmful, through interaction with indoor ozone (Weschler and Shields, 1999).

Although the existing literature supports the use of exposed wood surfaces in buildings under normal conditions, exposure to moisture may lead to microbial overgrowth and increased VOC emissions, which could have unintended negative health consequences. At moisture content (MC) levels above 30%, wood is more susceptible to fungal degradation (Cappellazzi et al., 2020), although mould growth can occur immediately at RH above 70–75% (Viitanen and Ojanen, 2007) or wood MC above 15% (Olsson, 2020). Despite the numerous positive characteristics of mass timber construction, architects and developers have encountered market barriers related to growth of mold and potential for biological deterioration in response to wetting (Mjörnell and Olsson, 2019). A number of studies have investigated the consequences of exposure to weather during construction of mass timber buildings (Kordziel et al., 2019, 2020; Kukk et al., 2022; Mjörnell and Olsson, 2019; Riggio et al., 2019; Schmidt et al., 2019), however, few studies have examined change in microbial communities on wetted mass timber under post-occupancy conditions, as might occur during unpredicted water intrusion (e.g., plumbing leak). Research to date has also focused primarily on fungal growth (Lepage et al., 2019; Pasanen et al., 2000; Wang et al., 2018), with less attention paid to bacterial taxa and little to no assessment of archaeal or viral taxa.

The goal of this research is to examine how physical and chemical qualities, including associated VOCs, of coated and uncoated CLT affect surface microbial communities under dry and several different wetting scenarios representing a range of duration and severity of moisture exposure that might be encountered in occupied buildings. We asked the following research questions:

1. Does wetting impact VOC emissions from coated or uncoated CLT blocks?
2. Does wetting impact microbial abundance, diversity, or composition?
3. Are VOC emissions from coated or uncoated CLT blocks associated with microbial abundance, diversity, or composition?

## Materials and methods

### Overview

**Figure 1:**
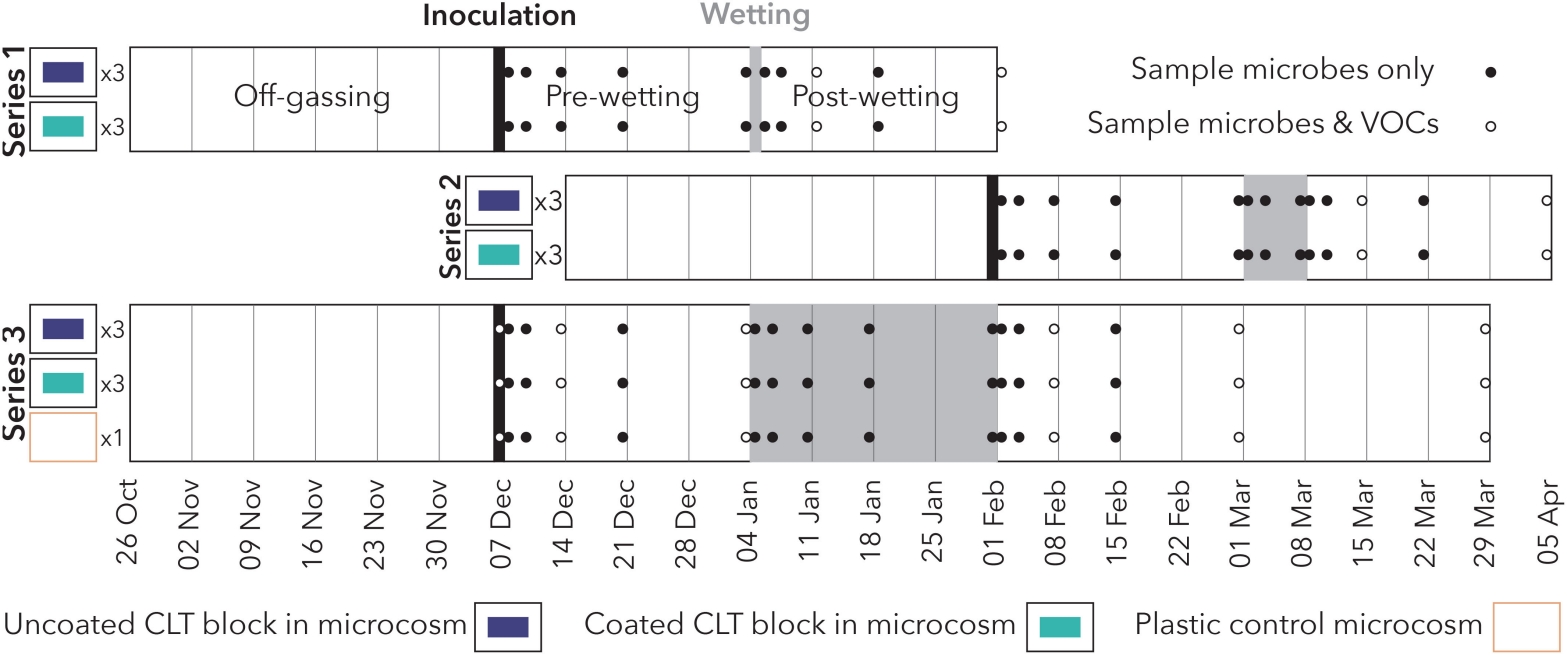
Experimental design of the study, comprising three series with varying wetting durations. Series 1 and 2 occurred sequentially using the same (disinfected) microcosms, while Series 3 occurred in parallel.

This experiment took place at the Institute for Health in the Built Environment at the University of Oregon (Eugene, Oregon, USA) between October 2020 and April 2021. We assessed hygienic and moisture performance of coated and uncoated CLT by examining VOCs and surface microbial communities before, during, and after wetting events of varying duration. Microbial and VOC samples from three replicates of coated and uncoated CLT blocks, nested within three series representing different wetting durations, were collected over the 4-month period (**Figure 1**).

- Series 1 short-duration wetting (20-minute spray of tap water on a single day).
- Series 2 medium-duration wetting (20-minute spray of tap water every day for 1 week) conducted immediately after Series 1 using the same microcosms but different CLT samples.
- Series 3 long-duration wetting (20-minute spray of tap water every day for 4 weeks) conducted in parallel with Series 1 and 2.

Each CLT block was placed in an ethanol-disinfected microcosm to eliminate contamination from other sources while controlling ventilation, temperature and humidity conditions. The experiment was broken into four phases: CLT coating and off-gassing, initial colonization under dry conditions, a wetting period of variable duration according to series, and a post-wetting period. The main objectives of this experimental design were to: 1) assess how wetting affects microbial abundance, diversity, and composition on coated and uncoated CLT surfaces over time; 2) assess how wetting affects VOC emissions from coated and uncoated CLT surfaces over time; and 3) assess how VOC emissions are related to microbial abundance, diversity, and composition.

### CLT block preparation and microcosm setup

A 122 × 244 × 10 cm (48 × 96 × 4 in) three-ply spruce pine fir (SPF) CLT panel was produced from sustainably forested wood sourced in the Pacific Northwest by Vaagen Timbers using melamine urea formaldehyde (MUF) adhesive. Eighteen identical 28 × 46 × 10 cm (12 × 1 8× 4 in) CLT blocks were cut with a five-axis CNC fabrication machine (Uniteam UT9) at the A.A. “Red” Emmerson Advanced Wood Products Lab at Oregon State University (OSU). Half of the blocks received two coats of Sansin KP-12W Protective Undercoat on all surfaces. This product is an aqueous, low-VOC wood undercoat formulated to repel moisture and reduce water absorption. According to the KP-12W Safety Data Sheet, the product contains 3-iodo-2-propynyl butylcarbamate (CAS#: 55406-53-6), 2-butoxyethanol (CAS#: 111-76-2), ammonium hydroxide (CAS#: 1336-21-6), and 2-amino-2-methyl-1-propanol (CAS#: 124-68-5). 3-Iodo-2-propynyl butylcarbamate is a widely-used fungicide. The first coat was applied to the unfinished top and sides of each block, allowed to dry for six hours, followed by the second coat, then the blocks were flipped and coatings similarly applied to the final surfaces. Blocks were disinfected with ethanol, placed in individual microcosms, and microcosm air flushed with a high air exchange rate of at least 140 air changes per hour (ACH) for six weeks during the off-gassing period. Pin-type moisture meters (Delmhorst Instrument Company BD-2100) were installed in the center of the upward-facing surface of each CLT block to measure wood moisture content (MC) at 6.35, 12.70, and 19.05 mm (0.25, 0.5 and 0.75 in) below the surface. Pins were coated on all exposed metal surfaces, except the tips, to ensure measurements accurately reflected the correct depth. They were hammered in through pre-drilled plastic blocks that allowed for constant depth and spacing without pre-drilling of wood, which could allow moisture to penetrate through the hole itself. Plastic blocks were removed prior to wiring the pins through junction blocks. Silicone was applied immediately around the pin to provide an additional barrier against moisture penetration through the hole.

**Figure 2:**
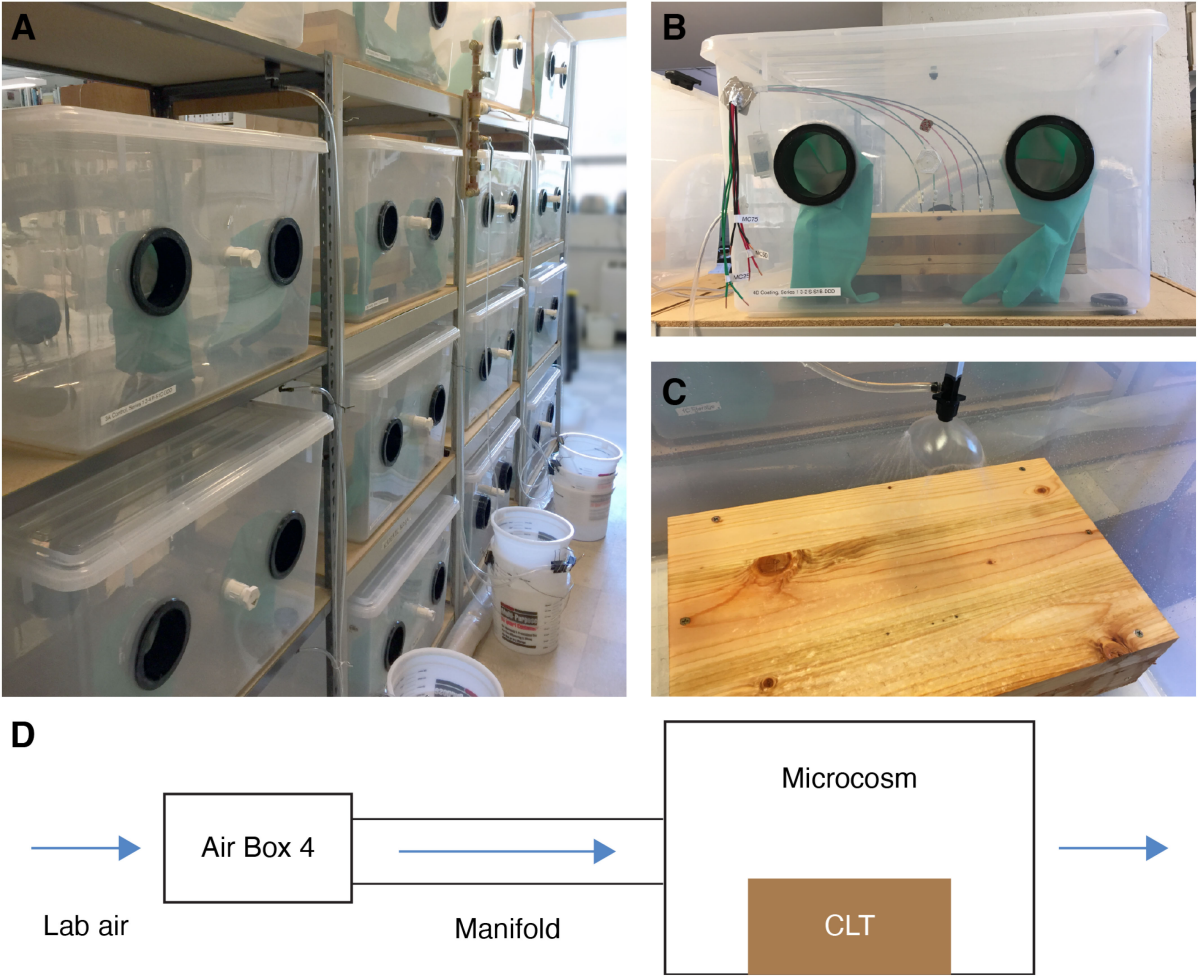
Photo of assembled microcosm array **(A)**, individual microcosm containing CLT block with moisture sensors **(B)**, wetting spray pattern **(C)**, and schematic of air filtration and delivery system **(D)**.

Microcosms were designed to eliminate contamination from other sources while controlling ventilation, temperature, and humidity conditions (**Figure 2**), similar to our previous experiment (Mhuireach et al., 2021). CLT blocks were placed on ABS pipe couplings to raise the sample about 40 mm above the microcosm floor, which prevented them from sitting in pooled water during the wetting events. Large (approximately 137.6 L) plastic storage bins were modified to accept nitrile chemistry gloves (ULINE S-19714-L) on the outward-facing side for sample collection, an access port for sampling swabs, a VOC sampling port, a ventilation duct supplying charcoal-filtered (Air Box 4 2000 CFM, Stealth edition) air on the opposite side, an exhaust air port, tubing for the irrigation emitters (Raindrip 153000) used to simulate wetting events, a water drain, and moisture meter wires. Each microcosm was sealed airtight and balanced at 2 to 3 Pa of positive air pressure, equivalent to 4 to 5 ACH in the sealed microcosms, with exhaust air exiting through the designated port. We used this airflow rate because, according to ASHRAE Standard 1702021 (ASHRAE, 2021), typical outpatient spaces require 2–3 ACH, while inpatient spaces, such as exam, treatment, and general patient rooms, require 4–6 ACH. Environmental conditions inside each microcosm (temperature, relative humidity, differential pressure) were monitored at 5-minute intervals with a HOBO data logger (Onset UX100).

### Inoculation, wetting, and sample collection

After the offgassing period, blocks were inoculated with a mock community containing *Escherichia coli*, *Pseudomonas fluorescens*, *Staphylococcus aureus*, *Salmonella typhimirium*, and *Staphylococcus epidermidis*, which are human-associated bacteria commonly encountered indoors. To create the mock community, each organism was incubated overnight on an LB agar plate. Following overnight incubation, one colony from each plate was transferred to a 15 mL conical tube containing LB broth and again incubated overnight. Successful cultures were centrifuged to pellet the cells, supernatant liquid was carefully removed, and 250 µL of sterile 1× PBS buffer solution was added. Cultures were then combined into a single tube and mixed thoroughly. Each block received 10 µL of this mock community suspension, which was applied across the entire wood surface with a sterilized spreader. The mock community was also applied to a control microcosm, which was identical to those used for the experiment, but did not contain a CLT block and was not subjected to the wetting event.

During the first phase of each series, blocks remained dry inside their respective microcosms to allow ecological succession of the mock community to stabilize. In this initial establishment period, microbial samples were collected immediately after inoculation and then 1, 3, 7, 14, and 28 days after being placed in the microcosms to characterize microbial survival, growth, and compositional change on dry wood and the plastic control microcosm. Nylon-flocked swabs (Copan Diagnostics) saturated with a 1× phosphate-buffered saline (PBS) solution were used to collect all microbial samples. Each block was sampled by moving the swab in multiple “S” patterns across the entire surface, while rotating the swab tip to ensure adequate biomass; a similar procedure was used to collect samples from the plastic bottom surface of the control microcosm.

For the wetting events, blocks were subjected to a continuous metered spray of untreated tap water for 20 min per day, a total average dose of 8.45 L (**Supplementary Figure S1**). A first flush of water was performed prior to each wetting event—water was purged for three minutes at the sink and for one minute from the sink to a 10 psi pressure regulator. Following the first flush protocol, water was passed through a manifold of needle valves to ensure equivalent flow rates to each irrigation emitter. After each wetting event, water was drained through the microcosm drain ports into containers for measurement. During Series 2, lab staff were unable to perform the wetting event for two days due to illness, thus this series was extended by one day. A single day of wetting was also missed during Series 3. The plastic control box did not undergo wetting. Tap water was expected to contain a variety of microorganisms, thus several samples were collected over the course of the experiment to identify which microbial taxa might be contributed to the surfaces of CLT blocks from the wetting event versus which taxa increased or decreased in relative abundance due to greater moisture availability after wetting.

Microbial samples were collected immediately after each wetting event and 1, 3, 7, 14, and 28 days later, following the same swabbing protocol as described above. An additional timepoint (post-wetting day 56) was sampled for Series 3 to assess whether the longer wetting duration impacted wood moisture content or microbial community composition over a longer time period. All microbial samples were immediately placed in a −20 °C freezer and stored until processing at the Biology and the Built Environment (BioBE) Center at the University of Oregon (UO) in Eugene, Oregon.

For VOC sampling, three technical replicates were collected from each microcosm and incoming laboratory air. Field blanks were also collected to assess potential VOC contamination from the researcher, transport, storage, or other experiment activities. Incoming laboratory air samples were used to determine initial concentration (C_0_) of identified VOCs directly after filtration and calculate emission rate from the materials. Samples were collected at two timepoints (**Figure 1**; 1 week and 4 weeks post-wetting) for Series 1 and Series 2, and six timepoints (**Figure 1**; immediately post-inoculation, 1 week and 4 weeks post-inoculation, and 1 week, 4 weeks, and 8 weeks post-wetting) for Series 3.

VOC collection protocols were identical to our prior experiment (Mhuireach et al., 2021). Briefly, samples were collected in glass sorbent tubes (PerkinElmer #N9307008) packed with 180 mg of Carbotrap B followed by 70 mg of Carboxen 1000 (Pankow et al., 1998) using a portable sampling pump (Universal PCXR8, SKC Inc., USA). Tubes were conditioned prior to each sampling timepoint, sealed with stainless steel Swagelok endcaps fitted with PTFE ferrules, and stored in plastic resealable bags at −6 °C prior to sample collection. All sampling and analysis of sorbent tubes occurred within a month after conditioning. The sampling was performed at a flow rate of 50 mL/min for 60 min with a total sample volume of 3 L for each sample. Two pumps were used for these experiments and the flow of each pump was measured each day (average of 15 measurements for each pump) using a primary flow calibrator (Gilian Gilibrator 2). After sampling, the sorbent tubes were capped and stored in two plastic resealable bags at −4 °C until analysis.

### VOC analysis

The samples were analyzed using an Absorption/Thermal Desorption (ATD) instrument (PerkinElmer TurboMatrix 650) connected to a gas chromatograph (model 7890 A, Agilent Technologies) with a DB-VRX column (60 m length × 0.25 mm i.d. × 1.4 µm film thickness, Agilent J&W) coupled to a mass selective detector (model 5975 C, Agilent Technologies). Each sample was desorbed at 300 °C for 10 minutes and all compounds were concentrated into a cold trap at −30 °C. Samples were then injected in a split/splitless injector maintained at 180°C. The injector was in split mode with a split flow of 2.76 mL/min. Helium was used as the carrier gas at a constant flow of 0.92 mL/min. The oven temperature started at 45 °C for 10 minutes, then increased by 12 °C/minute until reaching 190°C, after which it was maintained isothermal for 2 minutes. The temperature was raised again at 6 °C/min until reaching 240°C, kept isothermal for 5 minutes, and finally decreased at a rate of 10°C/min until reaching 210 °C. The mass spectrometry (MS) conditions were: transfer line at 230 °C, ion source at 250 °C and EI voltage at 70 eV. Data were recorded in full scan mode (m/z range: 34–400 amu).

Compounds were identified on the basis of their mass spectra and the injection of standards. The mass spectra were compared with those from two databases: National Institute of Standards and Technology (NIST) Mass Spectral Database 2008 (NIST08) and W8N08 library (John Wiley & Sons, Inc., USA). For visualization purposes, compounds were classified by putative sources (**Supplementary Table S1**), according to the National Center for Biotechnology Information (NCBI) PubChem database.

Quantification was achieved with five-point external calibration using a TO-15 gas mixture containing a representative mix of VOCs (65 component) from Linde (Alpha, NJ, USA) certified to ± 5% accuracy allowing for the identification and quantification of compounds. To verify thermodesorption and analysis efficiency and to obtain relative concentrations for those compounds lacking standards, four internal standards were also injected in each sample. Masses quantified on field blanks were removed from all the samples (coated and uncoated CLT blocks, plastic control microcosm, inlet air, and lab air). Emissions from the control microcosm were subtracted from calculated emission rates for microcosms containing CLT blocks; this allowed us to obtain VOCs emitted by the materials and the microorganisms inhabiting them. To calculate emission rates, we assumed steady state conditions in the microcosms, as they were in operation for approximately four months. A mass balance written on each chamber, assuming constant chamber volume, flow rate during a given sampling period, and emission rate is shown in Equation 1:


(1) $$dC/dt=\lambda C_{0}-\lambda C+E/V$$

where:

V = Volume of the chamber minus volume of the material (m^3^)
C = Concentration of the compound in the chamber (µg/m^3^)
C_0_ = Concentration of the compound in inlet air (µg/m^3^)
Q = Flow rate (m^3^/h)
*λ* = Exchange rate (h^-1^) = Q/V
E = Emission rate (µg/h)

In the case of a steady state, *dC*/*dt* = 0 and Equation 1 becomes:


(2) $$0=\lambda C_{0}-\lambda C+E/V$$

From Equation 2 we obtain the emission rate with Equation 3:


(3) $$E=\lambda (C-C_{0})*V$$

### Microbial analysis

#### Genomic material preparation

Tubes containing swab tips and PBS were vortexed briefly, then the swabs tips were removed, leaving the PBS containing genomic DNA. Samples were subdivided into two equal aliquots, one of which was treated with propidium monoazide (PMA) to discriminate between viable and nonviable DNA (Fittipaldi et al., 2012). After PMA treatment, genomic DNA was extracted from samples using MagMAX Microbiome Ultra Nucleic Acid Isolation Kit (ThermoFisher A42357) following manufacturer’s protocol.

#### Quantitative PCR

Absolute bacterial abundance in both PMA-treated and untreated aliquots was quantified using real-time quantitative polymerase chain reaction (RT-qPCR). Primers used were ABS Total Bacteria F SYBR Primer 5`-GTGSTGCAYGGYTGTCGTCA-3` and ABS Total Bacteria R SYBR Primer 5`-ACGTCRTCCMCACCTTCCTC-3`, which target the *E. coli* 16S regions 1048–1067 and 1175–1194, respectively, for a broad-range “universal” assay of bacterial cell counts (Maeda et al., 2003). Assays were performed in triplicate using 10 µL reaction volumes for experimental samples, as well as positive, negative, and no-template controls, using the following reaction mixture: 5 µL Luna Universal qPCR Mastermix (New England Biolabs #M3003), 0.25 µL forward primer, 0.25 µL reverse primer, 3.5 µL PCR-grade water, and 1 µL DNA template. Thermocycling conditions were programmed as follows: initial denaturation for 2 min at 50 °C, 2 min at 95°C; 40 cycles of 15 s at 95°C, 15 s at 60°C, and 60 s at 72°C; followed by a melt curve in the range of 60°C to 95°C. Serial dilutions of 16S Artificial DNA Standard (1.77 × 10^8^ genome copies per microlitre) were used to generate standard curves. The resulting genome copy estimates were used as a proxy for absolute bacterial abundance.

#### Shotgun metagenomics

Shallow shotgun metagenomics was utilized to generate taxonomic and functional data regarding the archaeal, bacterial, fungal, and viral communities. Genomic libraries were constructed from the PMA-treated samples using a LabCyte Echo Liquid Handler to perform miniaturized reactions with Nextera XT DNA Library Preparation Kit (Illumina, San Diego, CA, USA) at Genomics & Cell Characterization Core Facility (GC3F) at UO (Eugene, OR, USA). Samples had extremely low biomass, therefore, replicates of each material/timepoint were pooled together prior to library prep and sequencing. Qubit dsDNA high sensitivity quantitative assay was used to quantify DNA and libraries were pooled to 2 nm. Paired-end sequencing (2 × 150) was performed in a single Illumina NovaSeq (S4 300 Cycle) run on an at GC3F.

We used FastQC (Andrews, 2010) to examine read quality of raw sequences prior to trimming adapters and removing contaminants, including PhiX and human sequences, with BBDuk (Bushnell, 2021) using suggested parameters (ktrim=r k=23 mink=11 hdist=1 tpe tbo). Taxonomic classification of clean unassembled reads was performed with Kraken 2 (Wood et al., 2019), which uses exact kmer matching, against the standard database containing NCBI taxonomic information, as well as the complete bacterial, fungal, viral, and archaeal genomes in RefSeq (O’Leary et al., 2016). The resulting Kraken 2 report was converted into a BIOM table for later use in R using the kraken-biom tool (Dabdoub, 2021). We used the R package phyloseq to import the Kraken 2 BIOM table into R (McMurdie and Holmes, 2013). None of the negative control samples contained any reads, therefore we were unable to filter for potential laboratory contaminants. Control samples and experimental samples below our minimum threshold of 4,000,000 reads were removed prior to downstream analysis.

Clean reads were coassembled with MegaHIT (Li et al., 2015) using preset -metalarge, resulting in an average of 96,806 contigs. Open reading frames and total protein sequences were predicted from coassembled contigs using Prodigal (Hyatt et al., 2010). Antibiotic resistance features were annotated using hmmscan from HMMER3 (Eddy, 2011) against the full ResFams database, which provides a curated list of protein families associated with antibiotic resistance (Gibson et al., 2015).

### Statistical analyses

We conducted all analyses and visualizations in the R statistical computing environment, relying especially on the ggplot2 and patchwork packages to construct plots and figures (Pedersen, 2022; Wickham, 2016).

Environmental conditions were summarized by microcosm and sampling day prior to analysis. We used generalized least squares (GLS) to model the association of surface coating with environmental conditions (temperature, RH) and wood MC. In general, we followed Pinheiro and Bates (2000) to conduct analysis of response profiles using function gls from the nlme package (Pinheiro et al., 2023), with covariance structure set to corAR1 to adjust for temporal autocorrelation between data points.

Antedependence (AD) models, also known as Markov models, were employed to analyze changes in VOC concentration and emission rate and bacterial absolute abundance, as AD models can parsimoniously accommodate nonstationary time-dependent data (Núñez-Antón and Zimmerman, 2000). We used package mmrm and described the covariance structure with ‘adh’—AD models with heterogenous covariance (different within-subject variances). Estimated marginal means were computed with emmeans using Satterthwaite adjusted degrees of freedom and restricted maximum likelihood (REML) estimation. VOC technical replicates were averaged prior to statistical analysis; one VOC sample from a coated CLT block on Day 112 was an outlier and was removed prior to averaging.

We performed variance-stabilizing transformation, as implemented in DESeq2 (Love et al., 2014), to address differences in sample library sizes prior to beta diversity and differential abundance analysis. Compositional dissimilarity between samples was computed using the Morisita-Horn distance; these values were then used in permutational multivariate analysis of variance (PERMANOVA), as implemented by the adonis2 function in vegan (Oksanen et al., 2018), to assess the importance of sampling period (before, during, or after wetting), series, and surface type in driving microbial community structure. We next split the data by sampling period and microbial group (archaea, bacteria, fungi, virus) and again performed PERMANOVA to examine whether series or surface type was more influential before, during, or after wetting and whether the response differed by microbial group. We tested for a relationship between microbial composition and VOC composition using Procrustes analysis (Lisboa et al., 2014).

To assess whether individual taxa were differentially abundant on coated or uncoated CLT blocks, we used generalized linear models (GLMs) based on the negative binomial distribution for the entire microbial community, as well as for archaea, bacteria, fungi, and virus separately. For this analysis, we used the deseq function in DESeq2 and report results from the default Wald hypothesis test.

Our *a priori* significance threshold was 0.05 and we adjusted for multiple testing using the Benjamini and Hochberg method (Benjamini and Hochberg, 1995).

## Results

### Environmental conditions and wood moisture content

Temperatures inside the microcosms ranged from 13.3 to 25.1 °C and generally tracked the temperature of the laboratory in which the microcosm array was housed (**Supplementary Figure S2**). The mean daily temperature was 21.5 °C and mean standard deviation was 0.2 °C. Microcosm temperature was not correlated with surface coating (GLS; t = -0.546, p = 0.585).

RH inside the microcosms also generally tracked that of the external laboratory, except during wetting periods (**Supplementary Figure S3**). Mean daily RH during pre- and post-wetting periods was 40.0%, with a standard deviation of 5.8%. During wetting periods, microcosm RH rose to a maximum of 98.6% while tap water was spraying; it dropped to a minimum of 29.9% during wetting periods while tap water was not spraying. The average standard deviation during wetting periods was 15.5%. RH inside microcosms was also not correlated with surface coating (GLS; t = -0.103, p = 0.918).

Prior to the wetting events, both uncoated and coated mass timber samples maintained relatively stable moisture content (MC) between 5 to 10%. The overall mean MC before wettng was 7.4%. Wood samples received an average of 8.5 L of water sprayed onto the top surface each day of their respective wetting events (**Supplementary Figure S1**).

**Figure 3:**
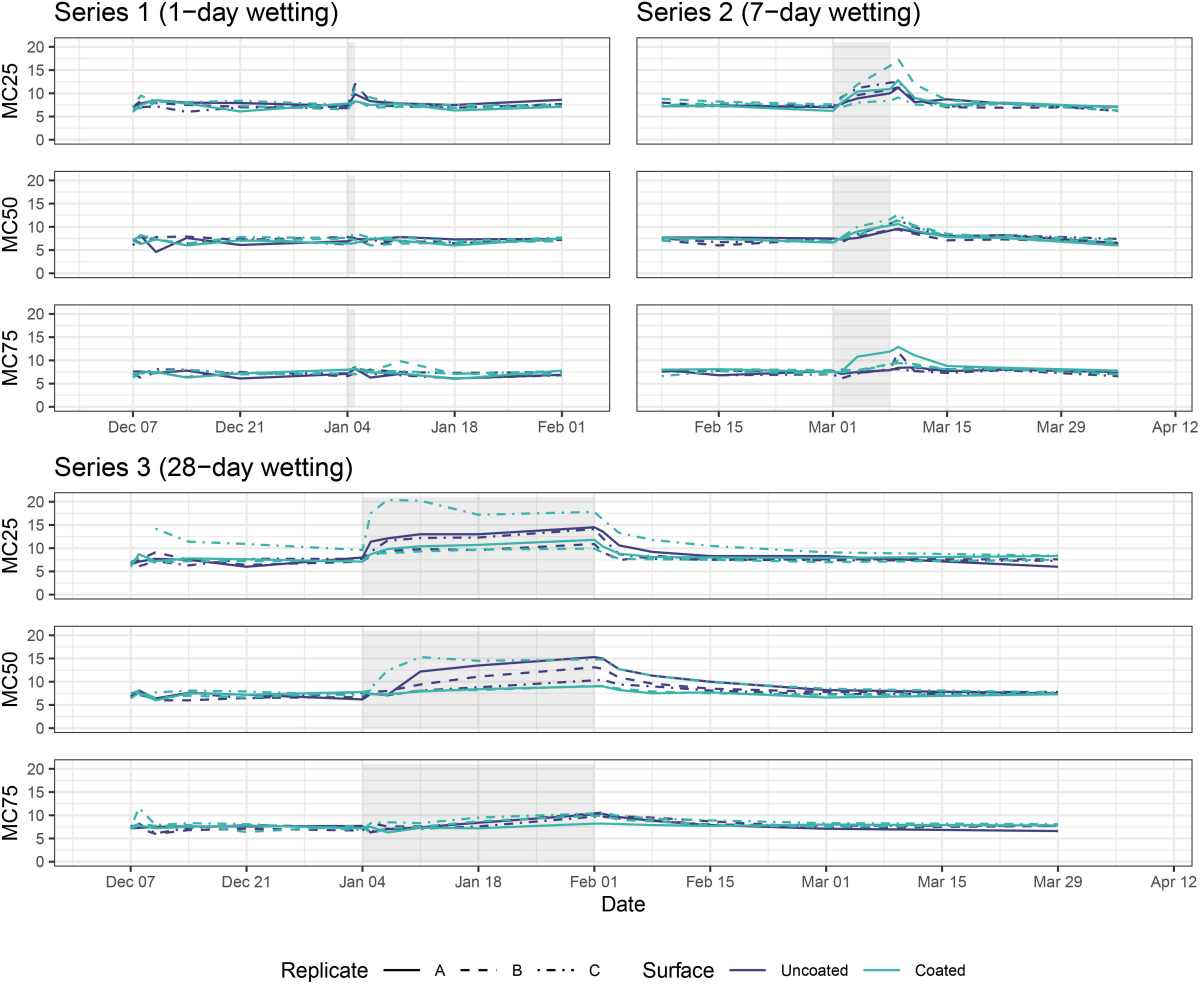
Moisture content of CLT blocks at 6.35 mm (0.25 in, top plot for each series), 12.70 mm (0.50 in, middle plot for each series), and 19.05 mm (0.75 in, bottom plot for each series) below top surface. Grey rectangles represent wetting periods.

For Series 1 (1-day wetting) samples, MC at all depths below the wood surface increased during and immediately after the wetting period before returning to baseline levels within 48 hours (**Figure 3**). Series 2 (7-day wetting) and Series 3 (28-day wetting) displayed similar patterns, but peaked at higher MC levels for all depths and took longer to return to baseline—about one week for Series 2 and about four weeks for Series 3. One caveat to the MC measurements is the potential for moisture to penetrate block surfaces through the pathway created by the pins themselves, thus these results are likely more pronounced than the actual MC would be in an unblemished CLT block. We found that MC25 (the shallowest pin) was correlated with coating only in the pre-wetting period (GLS; t = 2.8, p = 0.006), while MC50 was not correlated with coating in any periods, and MC75 was correlated with coating during all periods (GLS; pre-wetting: t = 2.45, p = 0.017; during wetting: t = 2.21, p = 0.031; post-wetting: t = 2.3, p = 0.022).

### VOCs

**Figure 4:**
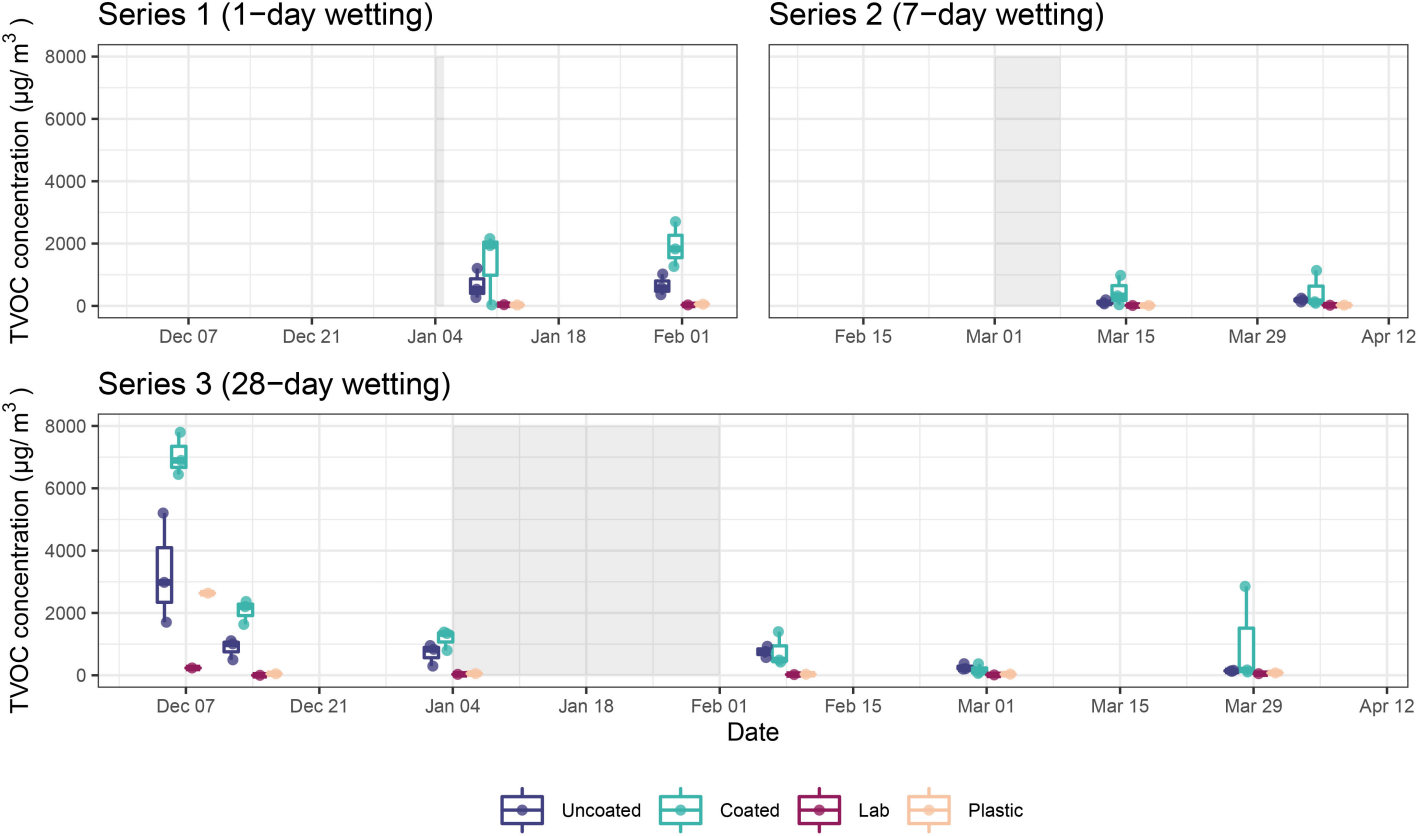
TVOC concentrations in microcosms containing coated and uncoated CLT blocks, as well as the plastic control microcosm and external laboratory for each series. Shaded areas represent wetting periods.

VOC samples from Series 1 (1-day wetting) and Series 2 (7-day wetting) were collected 7 and 28 days post-wetting, while samples were collected from Series 3 during the prewetting period, 7, 14, and 28 days after inoculation, as well as 7, 28, and 56 days postwetting. The highest TVOC concentration occurred immediately after microbial inoculation and decreased substantially over the course of the experiment (**Figure 4**). The elevated TVOC concentration in the plastic control box on Day 0 (07 December), suggests the inoculation process itself (either the microbes or the reagents) as a substantial source of VOCs, as the incoming laboratory air had negligible TVOC levels throughout the study. Aside from Day 0, TVOC concentration in the plastic control box was similar to incoming laboratory air and much lower than microcosms containing CLT blocks throughout the study.

In general, microcosms containing uncoated CLT blocks had lower TVOC concentration than those containing coated blocks (AD; t = 3.76, p = 0.004). This effect was especially pronounced earlier in the experiment, possibly due to continued off-gassing of the coatings. By Day 84 (01 Mar), TVOC concentration in both uncoated and coated samples decreased to less than 500 µg/m^3^, and by Day 112 (29 Mar) microcosm concentrations were not substantially higher than the incoming laboratory air or the plastic control box. One exception to this result was sample S.S3A.112, an outlier (which was removed prior to analysis) characterized by high levels of ethanol, acetone, cyclopentane, and 2-methylbutane.

**Figure 5:**
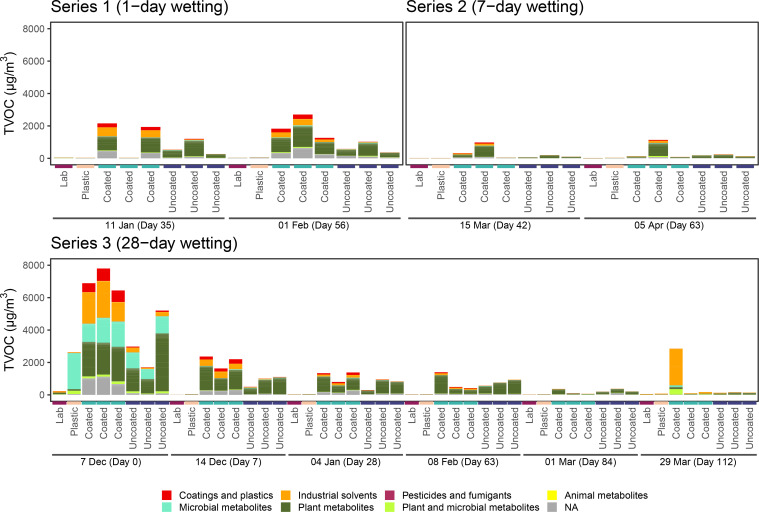
TVOC proposed sources for each wetting series. VOCs were classified using PubChem, as described in **Supplementary Table S1**. Bars represent individual samples. Within each series, bars are organized by sampling day, then by sample type. There are three replicates each day for coated and uncoated CLT blocks, and only a single sample each day for the plastic control microcosm and external laboratory.

TVOC composition in microcosms containing coated CLT blocks was dominated by VOCs putatively representing manmade sources, such as coatings and plastics manufacture and industrial solvents, as well as plant metabolites (**Figure 5**; **Supplementary Figure S4**). Uncoated CLT blocks, on the other hand, were mainly associated with plant metabolites, such as terpenes. The dominant monoterpene found in microcosms containing uncoated samples was *β*-phellandrene.

Overall, VOC emission rate followed a similar pattern to concentration inside the microcosms, with the notable exception that acetoin (3-hydroxy, 2-butanone) had a negative emission rate for all CLT samples on Day 0 (07 Dec), immediately after microbial inoculation (**Supplementary Figure S5**). The negative emission rates were due to our procedure of subtracting VOC concentrations observed in the plastic control microcosm from those observed in experimental microcosms, to find which VOCs were emitted by the CLT blocks versus the microorganisms themselves.

**Figure 6:**
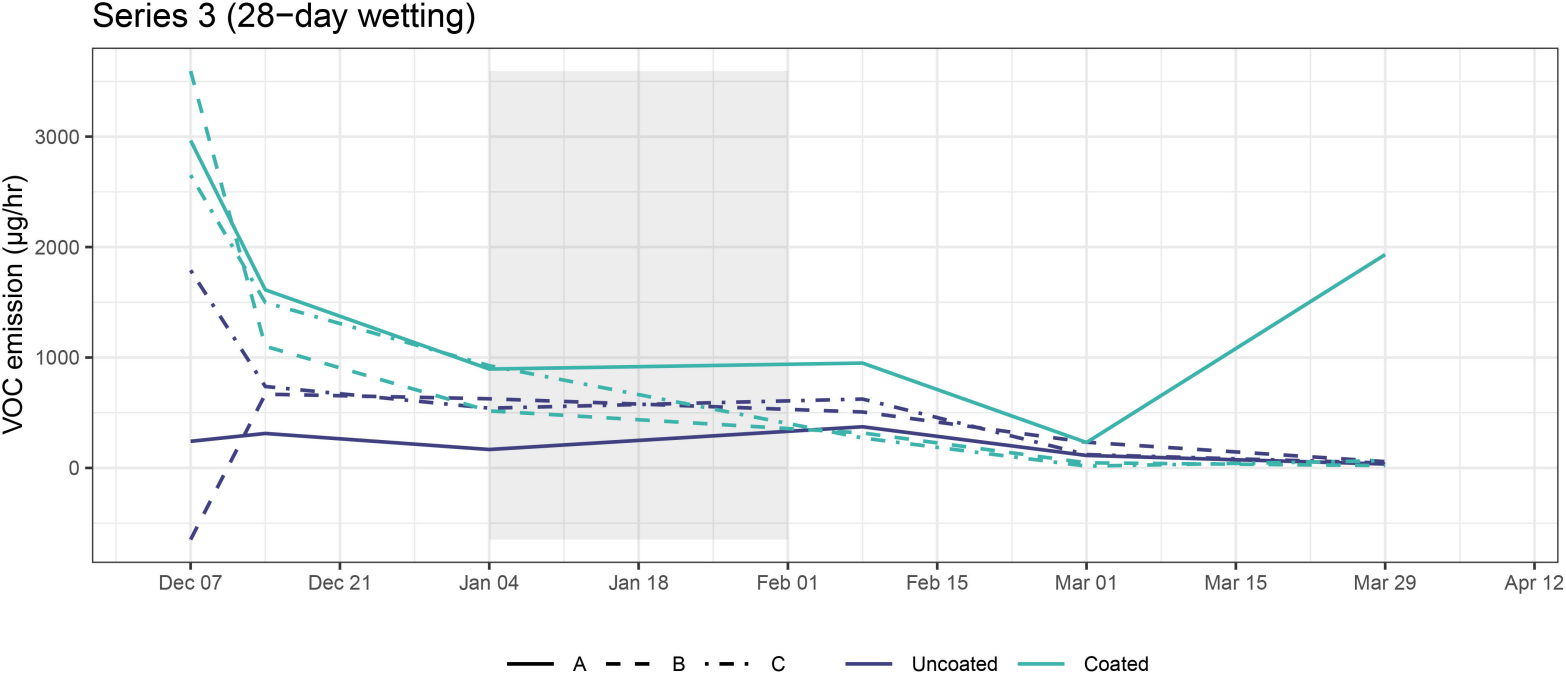
TVOC emission rates for coated and uncoated CLT blocks in Series 3. Shaded areas represent wetting periods.

Most CLT blocks had decreasing emissions over time (**Figure 6**). Interestingly, there was no statistically significant change in emissions before and after the wetting period (AD model; t = 0.92, p = 0.408), possibly indicating a slight increase in emissions that counteracted the overall decrease. This finding will be further interpreted in the Discussion.

### Microbial abundance

**Figure 7:**
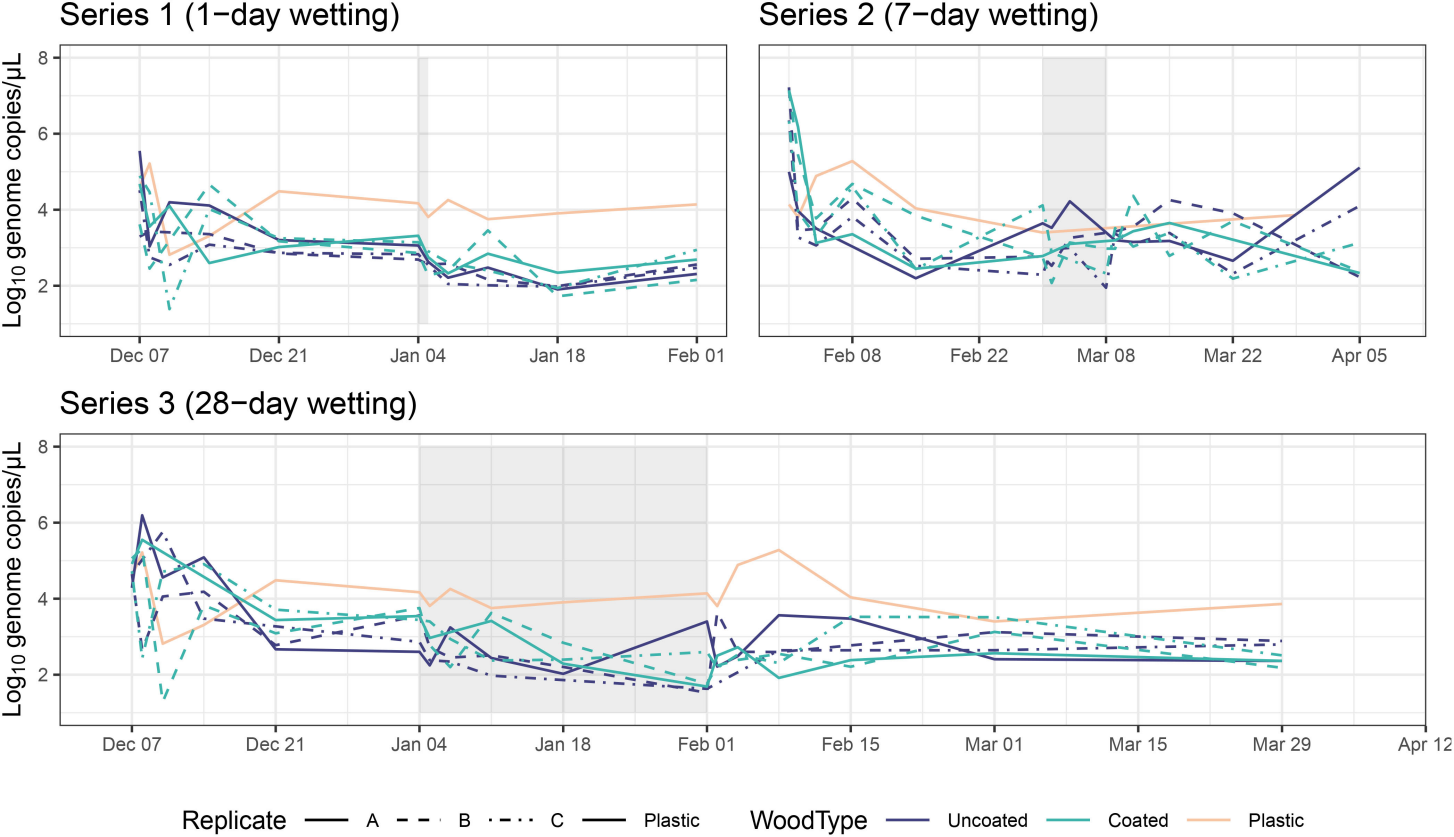
Viable bacterial abundance (estimated by gene copy numbers) over time for each series. Data for the plastic control box shown on all three series as a reference. Shaded areas represent wetting periods.

We observed a sharp decline in viable bacterial abundance on the first day after inoculation for most samples, followed by a moderate to pronounced bounce-back during the first week after the initial decline (**Figure 7**; total bacterial abundance over time shown in **Supplementary Figure S6** and viable:total ratios in **Supplementary Table S2**). Viable bacterial abundances appeared to be more or less stable for the remainder of the experiment, although individual microcosms experienced several dips and peaks, particularly related to the wetting periods. Viable bacterial abundance in Series 3, for which there were paired daily samples across the whole experiment, was negatively associated with RH (AD: t = -3.97, p = 0.004). Coating status also had a significant effect—uncoated CLT blocks generally had greater bacterial abundance than coated blocks (AD: t = 2.37, p = 0.036). Excluding the initial 14 days post-inoculation, samples from the plastic control box had an average of 21,118 more gene copies per µL than coated CLT blocks and 7,021 more gene copies per µL than uncoated CLT blocks.

### Microbial community diversity and composition

#### Overview

For microbial communities, a total of 2,770,870,677 raw paired-end reads were generated, averaging 25,420,832 reads per sample. After quality filtering, a total of 2,769,253,073 clean reads remained for use in downstream analyses (**Table 1**). Kraken 2 was able to classify 80% of the trimmed, quality filtered, unassembled reads.

**Table 1:** Total read counts, number of identified taxa, top most abundant taxon, and relative abundance (within their own group) of the top taxon in each microbial group.

Group	N.Reads	Obs.Taxa	Top.Taxon	Rel.Abund
Archaea	769306	356	Candidatus Halobonum	0.017
Bacteria	2210687524	7433	Pseudomonas	0.363
Fungi	8113383	83	Malassezia	0.445
Viruses	1346552	3444	Poxviridae (Family)	0.507
Total	2220916765	11316	Pseudomonas	0.362

Euryarchaeota (90.9%) was by far the most abundant phyla in archaeal communities overall, followed distantly by Crenarchaeota (4.3%), and Thaumarchaeota (3.2%). Bacterial communities were dominated by Proteobacteria (86.0%); Actinobacteria (9.0%) and Firmicutes (4.5%) were also relatively abundant. Ascomycota (52.9%), Basidiomycota (47.1%) and Microsporidia (0.04%) were the only fungal phyla observed in this study. The most abundant viral phyla were Nucleocytoviricota (53.4%), Uroviricota (19.4%), and Artverviricota (9.9%).

#### Alpha diversity

**Figure 8:**
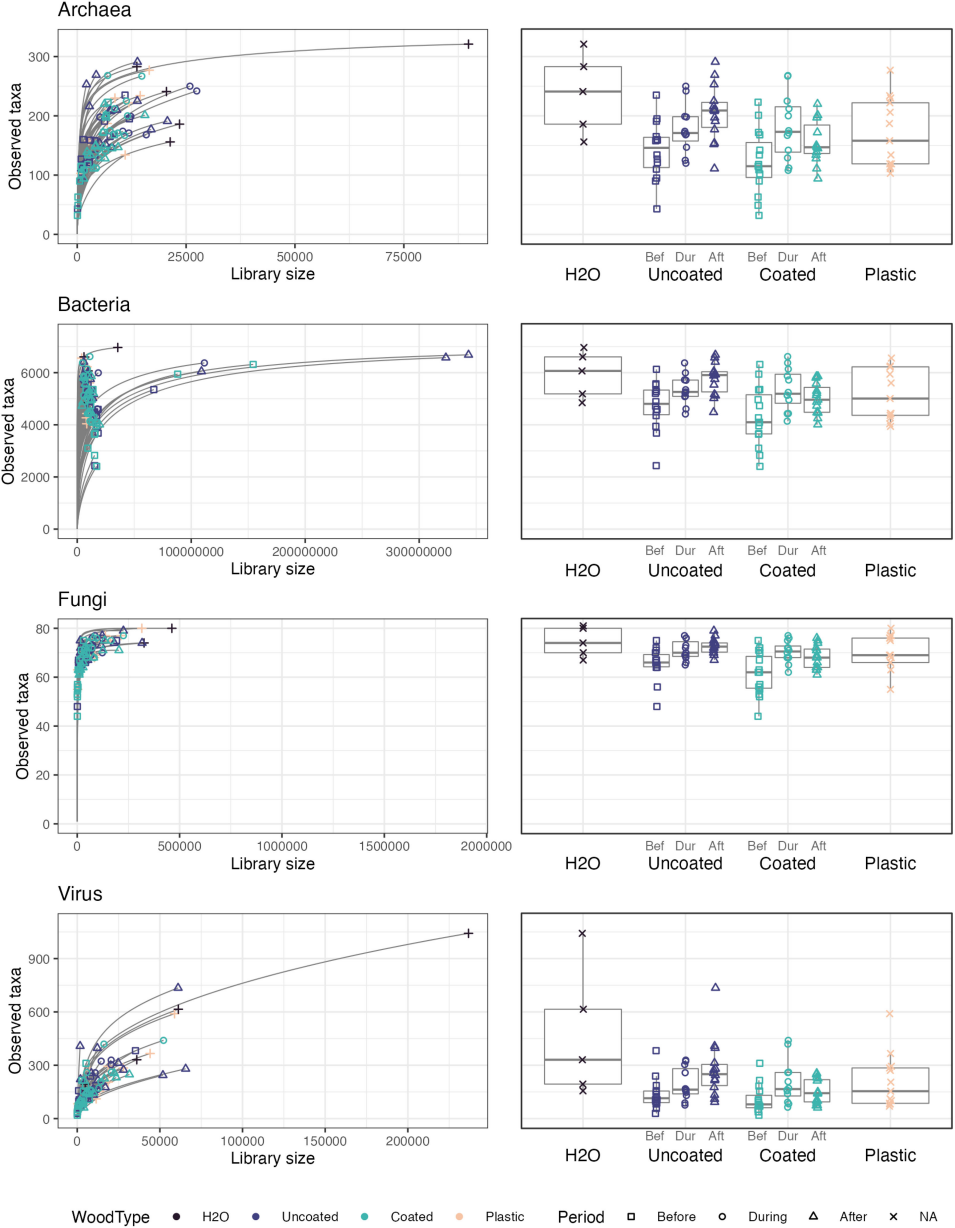
Rarefaction curves and numbers of observed taxa for each microbial domain.

Sampling depth was low across all microbial groups for most samples in this study (**Figure 8**), as was expected, given the extremely low biomass recovered from CLT surfaces. Thus, we share the data generated by this experiment, with the important caveat that much remains unknown about the structure of these microbial communities. We also did not perform statistical testing of the alpha diversity, because of this issue and general concerns about the validity of alpha diversity measures in microbiome studies (Willis, 2019).

A small handful of samples, including one tap water sample, had comparatively large library sizes, which tended to translate into greater numbers of observed taxa. Interestingly, for all microbial groups, uncoated CLT blocks showed a clear pattern of increasing richness by time period (before, during, after wetting), whereas coated CLT blocks had the greatest richness during wetting, followed by after wetting, then before wetting. Notably, coated blocks tended to have lower richness than uncoated blocks before and after wetting, although there appeared to be no difference in richness during the wetting period, likely due to the contribution of taxa from tap water. The plastic control box had an intermediate number of taxa, while tap water had higher richness than experimental samples, on average. These patterns were also reflected in estimated Shannon indices of alpha diversity, (**Supplementary Tables S3**–**S6**), which account for species evenness, as well as richness.

#### Taxonomic composition

**Figure 9:**
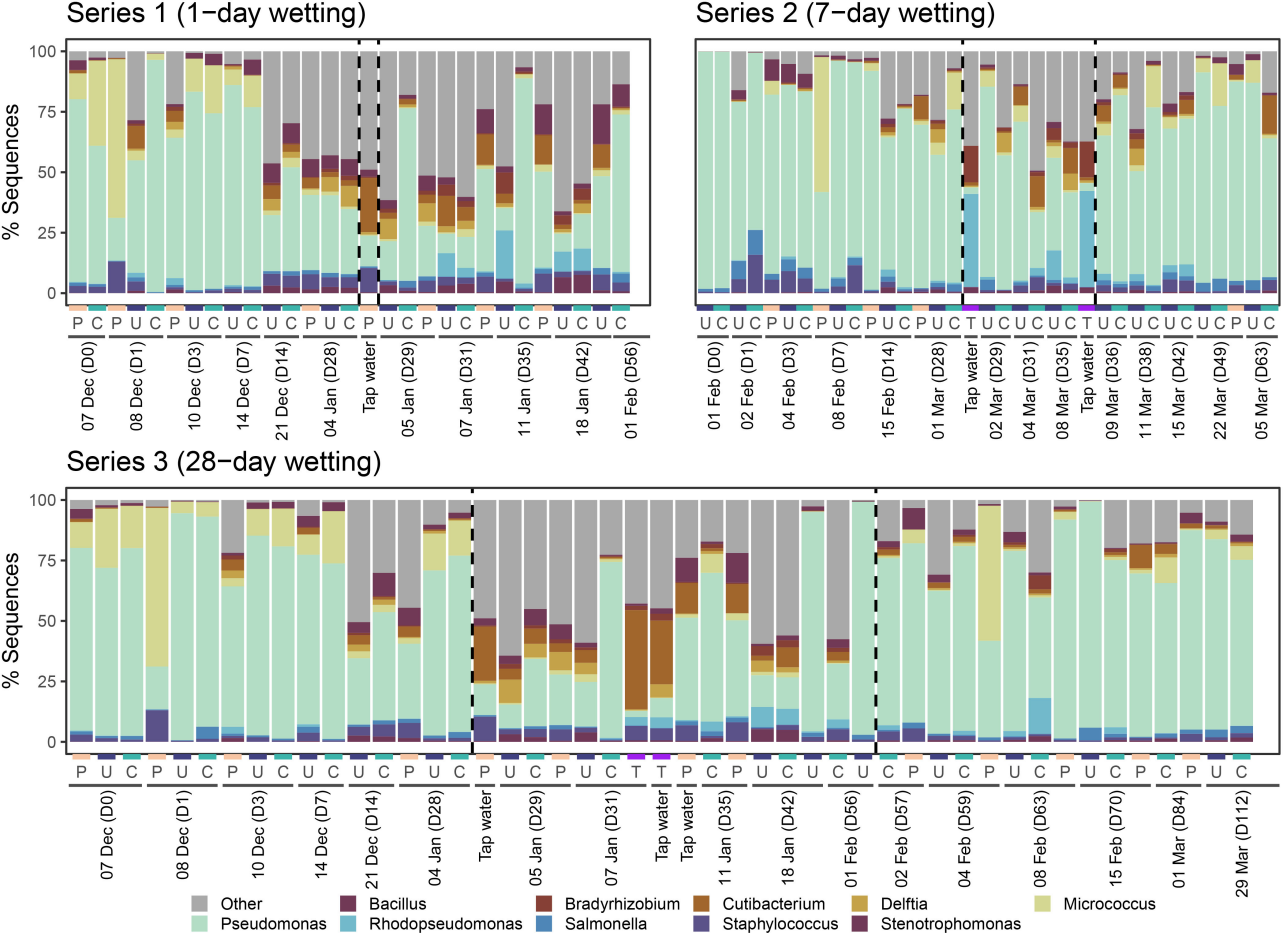
Relative abundance of the top 10 most abundant bacterial genera identified in each sample for each series. All other genera were aggregated into category “Other.” Samples are organized first by sampling day, then by sample type (P = Plastic, U = Uncoated, C = Coated, T = Tap water). Wetting period start and end days are indicated by dashed vertical lines. Tap water and control samples are shown in each series where relevant for reference.

Composition of the top 10 most abundant bacterial genera for each series are shown in **Figure 9**. *Pseudomonas*, *Staphylococcus*, and *Bacillus* (members of the top 10 most abundant taxa) were all part of the mock community with which CLT blocks were inoculated. Among bacterial taxa, *Pseudomonas* was dominant across all samples except tap water. Bacterial composition of water samples was quite different than coated, uncoated, and control samples. *Cutibacterium* and *Rhodopseudomonas* had higher relative abundance in tap water. The wetting period appeared to affect the composition of samples in Series 1 and 3, but did not seem to have as much impact in Series 2.

Regarding other microbial groups, we did not observe any striking patterns in archaeal abundance, although halophilic and methanogenic taxa were common among the top ten (**Supplementary Figure S7**). Fungal communities had high relative abundance of *Malassezia*, *Pyricularia*, and *Colletotrichum*, but also did not display any obvious patterns (**Supplementary Figure S8**). *Lessievirus* was abundant in samples from Series 1 and 3 on days 0–7, which were immediately after inoculation, yet to our knowledge this taxon was not present in the mock community. Similarly, between days 29–42, a number of Series 1 and 3 samples across all groups (coated, uncoated, plastic control) had high relative abundance of *Alphapapillomavirus* (**Supplementary Figure S9**). Since those days followed onset of the wetting periods, it might be thought that this virus was in the tap water, yet the tap water sample collected on January 4 had only a very low relative abundance of this taxon. Although this experiment took place during the COVID19 pandemic, we recovered no sequences identified as SARS-Cov-2.

#### Relative abundance patterns of mock community taxa

We examined relative abundance (with respect to only the bacterial component of each sample) patterns for specific taxa (*E. coli*, *P. fluorescens*, *S. aureus*, *S. epidermis*, *S. typhimirium*) that were in the original mock community. *E. coli* had a low relative abundance throughout the study (**Supplementary Figure S10**). It had higher relative abundance on the plastic control microcosm during the wetting periods in Series 3 when CLT blocks, but not the plastic control microcosm, were receiving tap water spray, possibly hinting that the water spray was washing bacterial cells off of the surfaces or making the surfaces otherwise inhospitable. This pattern was not seen in Series 2, however. *P. fluorescens* had very high relative abundance across all series and surface types during the majority of the study, but not in tap water samples (**Supplementary Figure S11**). Despite its high overall relative abundance, we were unable to discern any consistent pattern in the fluctuations from day to day. *S. aureus* had moderate relative abundance throughout most of the study (**Supplementary Figure S12**). There were several dramatic increases in its relative abundance on particular surface types and sampling days, notably on the plastic control microcosm on the day following inoculation and the day following onset of the wetting period for Series 1 and 3. *S. epidermis* also had a moderate relative abundance and its pattern over time was similar to that of *S. aureus*, but less pronounced (**Supplementary Figure S13**). *S. typhimirium* was not observed among the identified *Salmonella* taxa. One taxon could not be identified to the species level and had a relative abundance of 0.013, which may have been *S. typhimirium*.

#### Beta diversity

Initial data exploration showed that sampling period (before, during, or after wetting) was the most influential factor driving compositional dissimilarity for the entire microbial community (ADONIS: R^2^ = 0.104, p < 0.001), followed by series (R^2^ = 0.052, p = 0.003). Surface type did not appear to play a role at this level of analysis (R^2^ = 0.008, p = 0.759). We next split the data by period and microbial group to examine whether series or surface type was more influential before, during, or after wetting and whether the response differed by high-level taxonomic group.

On dry wood surfaces, prior to wetting, sampling day was a significant factor driving compositional similarity for all microbial groups (**Table 2**). This suggests that the weeks after inoculation experience community successional changes as different species populations fluctuate according to their ability to persist and thrive on the CLT blocks. For archaea, bacteria, and viruses, series was also influential. During the wetting period for each series, sampling day was an important factor for bacteria, fungi, and viruses; surface type was also important for viruses only. None of the factors were significant drivers of archaeal communities during wetting. After wetting, surface type was influential for all microbial groups and series was influential for bacteria, fungi, and viruses. No relationship between microbial composition and VOC composition was found using Procrustean association matrix analysis (Correlation = 0.348, p = 0.096).

**Table 2:** Results of PERMANOVA testing for each microbial group, separated by sampling period (before, during, after wetting).

Period	Variable	Arc.R2	Arc.P	Bac.R2	Bac.P	Fun.R2	Fun.P	Vir.R2	Vir.P
Before	Type	0.04	0.283	0.04	0.16	0.04	0.257	0.03	0.337
Before	Series	0.11	0.002***	0.14	0.004***	0.04	0.666	0.13	0.004***
Before	Day	0.06	0.036**	0.16	0.000***	0.19	0.002***	0.08	0.003***
During	Type	0.05	0.922	0.09	0.13	0.08	0.243	0.11	0.015**
During	Series	0.08	0.465	0.09	0.145	0.11	0.069*	0.08	0.136
During	Day	0.06	0.099*	0.07	0.034**	0.1	0.014**	0.07	0.004***
After	Type	0.07	0.022**	0.06	0.045**	0.07	0.022**	0.06	0.027**
After	Series	0.09	0.082*	0.14	0.001***	0.12	0.023**	0.1	0.033**
After	Day	0.05	0.087*	0.05	0.111	0.03	0.484	0.04	0.127

* p-value < 0.1, ** p-value < 0.05, *** p-value < 0.01.

#### Differential abundance

**Figure 10:**
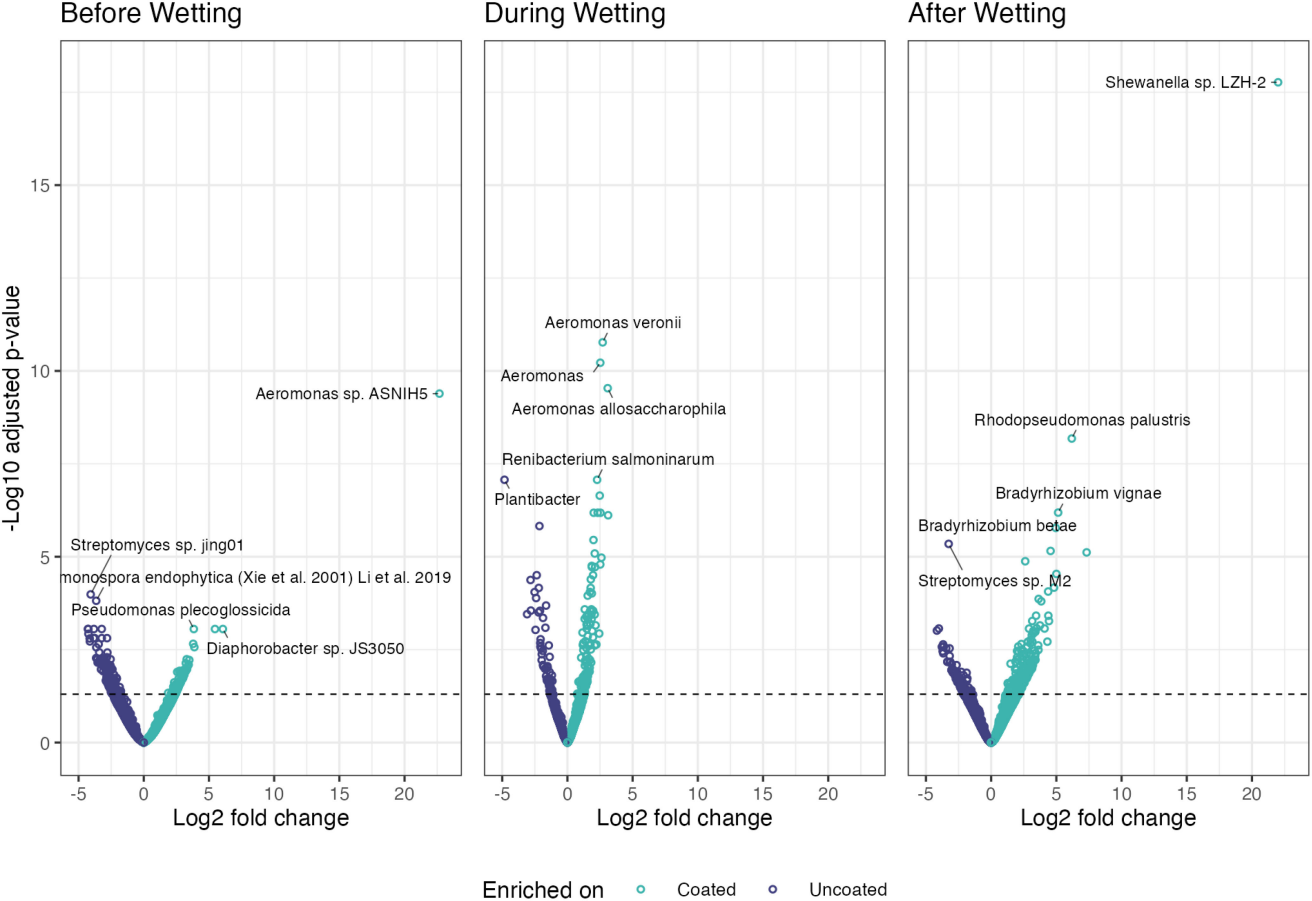
Enriched and depauperate taxa of all microbial groups across the three periods (before, during, after wetting).

Bacteria comprised the majority of differentially abundant taxa when comparing coated and uncoated CLT blocks before, during, after wetting (**Figure 10**). This is unsurprising, given that identified bacterial taxa outnumbered any other microbial group by at least an order of magnitude.

On dry wood surfaces, before wetting, no archaea or viruses were differentially abundant, fungal taxon (*Eremothecium gossypii*) was enriched on coated blocks, and bacterial taxa were enriched on coated blocks and bacterial taxa were enriched on uncoated blocks. During the wetting event, (*Halorussus* sp. *ZS-3*) archaeal taxon was enriched on coated blocks, while (*Halovivax ruber*, *Methanothermobacter* sp. THM-1, and *Geoglobus ahangari*) were enriched on uncoated blocks. For fungi, taxon (*Fusarium* sp.) was enriched on coated blocks and (*Sugiyamaella lignohabitans* and *Komagataella phaffii*) were enriched on uncoated blocks. No viral taxa were enriched on coated blocks during the wetting periods and only (*Fromanvirus Mycobacterium virus Switzer*) was enriched on uncoated blocks. After wetting, archaeal taxa (*Halorussus* sp. *ZS-3*, *Halosimplex pelagicum*, and *Thermofilum* sp. *3507LT*) were enriched on coated blocks and archaeal taxa were enriched on uncoated blocks; bacterial taxa were enriched on coated blocks and on uncoated blocks. No fungal or viral taxa were differentially abundant after wetting.

## Discussion

This study examined moisture content (MC), VOC emissions, and microbial communities on coated and uncoated CLT blocks before, during, and after wetting periods of varying duration. We found that average MC values during the pre-wetting period were similar to values reported by other studies for CLT panels during production and shipping (Kordziel et al., 2019), as well as to the equilibrium moisture content predicted by the 1999 Wood Handbook (Glass et al., 2014). However, MC in this study returned to baseline much more quickly than described in other work (Kordziel et al., 2019; Schmidt and Riggio, 2019; Schmidt et al., 2019; Wang et al., 2020), possibly due to the relatively high airflow of the microcosms, which were based off typical healthcare ventilation guidelines, or to the temperature and relative humidity of supply air coming from the external laboratory space. Mass timber in this study was exposed to airflow over its surface, allowing it to dry after wetting and return to baseline conditions. In the built environment, this may be similar to a floor/ceiling assembly in which the ceiling is mass timber exposed to the indoor environment. However, this study did not consider encapsulation of mass timber in an assembly, such as by gypsum board, insulation, acoustic or structural elements. In this scenario, drying would likely be prolonged. We also found that coating status was not correlated with MC during wetting, except for the deepest measuring pin (MC75). One explanation for this finding may be that moisture was able to penetrate into the wood via the hole drilled to insert the measuring pins. In particular, for Series 2 and Series 3, one of the coated CLT blocks tended to have higher MC than uncoated blocks. Although this observation did not agree with our *a priori* expectation that the coating would repel moisture, we did not exclude it as an outlier, since there were only three replicates.

Both uncoated and coated CLT blocks emitted more VOCs throughout this 119-day study than the plastic control box. As the coated blocks had higher VOC concentration during the first four weeks of the experiment, we suspect there was continued off-gassing from the coating application, which was completed six weeks prior to the experiment’s start. The age of the plastic control box was unknown, thus, it may have had a longer period since its manufacture to offgas, compared with the CLT blocks in this study. VOC emission rates from coated CLT blocks decreased by 99.1% and emission rates from uncoated CLT blocks decreased by 90.1% over the course of this 112-day study, following similar trends observed for other building materials (Huang and Haghighat, 2002). A study of Scots pine and Norway spruce blocks found that monoterpene emissions decreased 68–87% during one year of storage, and that rehydration of dry wood resulted in temporarily increased emissions (Muilu-Mäkelä et al., 2021). Similarly, in this experiment we noted a plateau of VOC emissions during the wetting period, which we interpreted as a slight increase in CLT emissions due to wetting.

Emission fluxes (the emission rates presented in **Supplementary Figure S5**, normalized by the projected area of test materials) are ~100 µg/(m^2^ h) for uncoated CLT and ~1000 µg/(m^2^ h) for coated CLT; these values are lower and similar to previosly presented emission fluxes for polymeric building materials (Yu and Crump, 1998). Since new construction typically takes many months, by the time the building is occupied, VOC emissions from structural materials are likely to be much lower than those coming from other sources, like equipment, furnishings, and the occupants themselves. One study estimated average human VOC emissions at 6.3 mg/hr/person (Tang et al., 2016). Formaldehyde was not included in our VOC analysis, due to our analysis methodology, which was aimed at exploring potential interactions between mass timber, microbial communities, and VOCs typically associated with natural wood, such as terpenes. In this experiment, terpenes comprised 62.2% of VOC emissions from uncoated CLT blocks and 18.8% from coated blocks, which were dominated by VOCs emitted by paints and solvents. Similarly, Alapieti et al. (2021) found that uncoated pinewood samples emitted large quantities of terpenes, while painted pinewood samples emitted primarily paint-associated compounds. Despite terpenes comprising a smaller proportion of the VOCs emitted by coated CLT blocks, the amount was numerically larger than the amount of terpenes emitted by uncoated blocks, which may be due to terpenes in the formulation of the coating.

We speculate that the high value of VOC emissions in the plastic control box immediately after inoculation (**Supplementary Figure S4**) may have come from the microorganisms in the mock community, since the most abundant VOC in that sample was 3-hydroxy-2-butanone (acetoin), with a concentration of 1663 µg/m^3^. There were also high levels of this compound in microcosms containing both coated and uncoated CLT blocks, but not in the incoming laboratory air, nor in noticeable quantities during the remainder of the experiment. Acetoin is produced by many bacteria, including *B. subtilis*, *S. aureus*, and *E. coli* (Camus et al., 2020; Petrov and Petrova, 2021; Xiao and Xu, 2007). A number of other compounds associated with the acetoin pathway, including 2,3-butanediol, 3-methylbutanoic acid, acetic acid, 2,3-butanedione, 2-butoxy-ethanol, and 2-methylbutanoic acid were also among the most abundant VOCs in this particular sample. 2,3-butanedione (diacetyl) and 2,3-butanediol (2,3-butylene glycol) are biological analogues of 3-hydroxy-2-butanone (acetoin), each of which represents a different oxidation level of the same “four-carbon skeleton” and may be alternatively produced during bacterial fermentation processes, depending on environmental conditions (Collins, 1972; Lu et al., 2016; Sharma and Noronha, 2014). It has been hypothesized that the relationship between acetoin production by some taxa (e.g., *Staphylococcus*) and acetoin consumption by other bacteria (e.g., *Pseudomonas*) may represent a form of trophic cooperation that benefits both taxa by providing an energy source for the non-producer and reducing toxic accumulation of acetoin for the producer (Camus et al., 2020). Acetoin can also act as a plant growth promoting compound, as demonstrated by studies of rhizobacteria, such as *B. subtilis* (Bennett et al., 2012).

All series exhibited instability in viable bacterial absolute abundance immediately after inoculation and during the wetting periods; abundance was more consistent during dry periods (with the exception of the post-wetting period in Series 2), possibly as a result of the hostility of the surface to alien microbes from a different source (e.g., inoculation broth, tapwater). Average daily RH (a proxy for wetting) was negatively associated with bacterial abundance in Series 3. During the dry periods before and after wetting, average daily RH in the microcosms was 36%, whereas during the wetting period the average daily RH rose to 62%. Previous studies have shown that bacteria living at surface-air interfaces tend to have lower survival rates at high RH (50–90%) compared with low RH (10–30%) (Stone et al., 2016; Turner and Salmonsen, 1973). Another possibility could be that increased VOC emissions from the CLT blocks could inhibit survival of bacteria. After accounting for the wetting period, uncoated CLT blocks generally had higher bacterial abundance than coated blocks. One ingredient in the coating (3-iodo-2-propynyl butylcarbamate) is known for its biocidal properties, which could explain this result. Wood blocks also had lower abundance than the single plastic control microcosm throughout the majority of the experiment, which could not be tested statistically due to lack of control replicates, but aligns with the preponderance of research showing that plastic surfaces typically allow greater recovery of microorganisms than wood surfaces (Koch et al., 2002; Milling et al., 2005).

The majority of samples submitted for shotgun metagenomics sequencing were characterized by very low biomass, possibly due to the difficulty of recovering microbial cells from wood surfaces, which has been observed in numerous other studies (e.g., Abrishami et al., 1994; Ak et al., 1994; Boursillon and Riethmüller, 2007; Coughenour, 2009; Da Costa et al., 2008; Greatorex et al., 2011; Hedge, 2015; Milling et al., 2005). With relatively simple original communities, until wetting periods, a high percentage (81%) of the trimmed, quality-filtered, unassembled reads were able to be classified. The bacterial mock community used for inoculation included taxa that are well-represented in NCBI RefSeq bacterial database, and bacteria comprised 99.5% of the identified community.

Uncoated CLT blocks displayed increasing richness by time period (before, during, after wetting), which may suggest that new microbial taxa were added to the surface community during wetting events and were able to persist. On the other hand, for coated CLT blocks, richness increased during wetting but then decreased again afterward, possibly indicating a more transient effect on diversity. Another possible explanation for increasing richness during wetting is that the tap water spray could wash off natural wood compounds that normally play a protective role against microbial degradation. Tap water had greater diversity than either coated or uncoated CLT, thus, the increase in diversity on CLT blocks during wetting periods was likely due to new microbial taxa being added to the surface community from tap water. Bacterial taxa observed in the tap water samples, particularly *Rhodopseudomonas* sp., were also observed in the CLT surface samples after wetting events, suggesting that these taxa were transferred to CLT from the tap water. *Rhodopseudomonas* is a member of the purple non-sulfur degrading group and is commonly found in anaerobic water, including wastewater, lakes, swamp, and marine bodies (Li et al., 2022). Influence of tap water application was not observed for archaea, fungi, or viruses, possibly due to the paucity of information about these groups of organisms in the NCBI RefSeq databases at the time of this analysis. We also found that the fungal taxon *Sugiyamaella lignohabitans*, which is commonly associated with decaying wood (Shi et al., 2021), was enriched on uncoated CLT blocks during the wetting periods.

A major limitation of this study was the very low biomass recovered from experimental samples, which led to pooling biological replicates and hampered our ability to draw conclusions supported by hypothesis testing. Prior to beginning the experiment we ran several brief pilot experiments to test our protocols and quantify the amount of recovered microbial DNA by qPCR. The pilot tests indicated that sufficient biomass could be recovered, given the amount of mock community stocks that the blocks were inoculated with. However, the pilot tests were very brief in comparison to the actual experiment, the quantity of bacterial cells contained in the mock community was not estimated, and the recovered DNA was not subjected to PMA treatment prior to qPCR. Thus, it is possible that much of the recovered DNA in the pilot may have been nonviable. We also note that the very low biomass recovery could be viewed as an interesting result of the study, rather than a limitation, since it demonstrates that the microbial inoculant is quickly drawn into the wood pores and cannot be recovered later. Another potential drawback of the study was the high microbial diversity of tap water, which resulted in some ambiguity regarding the fate of the original inoculant. Finally, due to the low biomass issue, we did not quantify the absolute abundance of fungi using qPCR, which would have allowed us to assess the degree to which fungicidal ingredients in the commercial coating mixture may have contributed to differences in fungal abundance.

## Conclusion

Architectural usage of CLT and other types of mass timber have increased recently, due to the perceived sustainability and other benefits of wood. However, concerns about the hygienic and moisture performance of exposed wood limit its use in certain applications, like healthcare. Answering our initial research questions, the results of this study suggested that: 1) wetting may cause a slight increase in VOC emissions, which is more pronounced in uncoated than coated CLT, that temporarily counter-acts a general decrease over time; 2) wetting periods tended to reduce abundance of viable bacterial cells on both coated and uncoated CLT surfaces, while also affecting microbial composition; and 3) VOC and microbial composition were not correlated, but the slight increase in VOC emissions associated with the wetting period may be related to the reduced bacterial abundance during the same period.

This study is the first, to our knowledge, to explore relationships between microbial communities inhabiting CLT surfaces and VOC emissions under dry and wetted conditions. Future research priorities should include expanded testing of different coating types, quantification of functional pathways that might relate VOC generation or degradation with microbial communities, and improved methods of collecting microbial samples from porous wood surfaces to ensure adequate biomass for metagenomic analysis.

## Data Availability

The original contributions presented in the study are publicly available. This data can be found here: NCBI Sequence Read Archive, accession PRJNA1242807.
