# Supplementary material for: Effects of wetting events on mass timber surface microbial communities and VOC emissions: implications for building operation and occupant well-being
Source: Front Microbiomes. 2025 Apr 9;4:1395519. doi: 10.3389/frmbi.2025.1395519 (PMC12993639; doi:10.3389/frmbi.2025.1395519)
Supplement: Supplementary file 1 [file DataSheet1.pdf]

Effects of wetting events on mass timber surface microbial communities and VOC emissions: Implications for building construction and occupant well-being

Gwynne Á. Mhuireach *[1], Susan Collins[2], Leslie Dietz[3], Patrick Finn Horve[4], Aurelie LaGuerre[5], Dale Northcutt[1], Jason Stenson[1], Kevin Van Den Wymelenberg[6], Elliott Gall[7], and Mark Fretz[1]

[1]**Institute for Health in the Built Environment, University of Oregon, Eugene, OR, USA**
[2]**Ginkgo Bioworks, Inc., Boston, MA, USA**
[3]**College of Engineering, Oregon State University, Corvallis, OR, USA**
[4]**Institute of Molecular Biology, University of Oregon, Eugene, OR, USA**
[5]**Salk Institute for Biological Studies, San Diego, CA, USA**
[6]**University of Nebraska–Lincoln, Lincoln, NE, USA**
[7]**Healthy Buildings Research Laboratory, Portland State University, Portland, OR, USA**

SUPPLEMENTAL INFORMATION

Table S1. Putative sources for VOCs identified in this study. Information was accessed from NCBI PubChem records June–September, 2023.

Compound	Putative.source
(-)-4-terpineol	Plant metabolites
(-)-borneol	Plant metabolites
(-)-Camphor	Plant metabolites
1-(2-methoxypropoxy)-2-propanol	Industrial solvents
1-Butanol	Microbial metabolites
1-heptanol	Microbial metabolites
1-Hexanol	Microbial metabolites
1-hydroxy-2-propanone	Plant and microbial metabolites
1-iodoethane	Unknown
1-methoxy-2-propanol	Industrial solvents
1-methyl-4-(1-methylethenyl)-benzene	Plant metabolites
1-octanol	Plant metabolites
1-pentanol	Microbial metabolites
1-penten-3-ol	Plant metabolites
1-Phenoxypropan-2-ol	Industrial solvents

Continued on next page

*Corresponding author

Continued from previous page

Compound	Putative.source
1-propanol	Microbial metabolites
1,1-dichloroethane	Industrial solvents
1,1-Dichloroethene	Industrial solvents
1,1,1-trichloroethane	Industrial solvents
1,1,2-trichloro-1,2,2-trifluoroethane	Industrial solvents
1,1,2-trichloroethane	Industrial solvents
1,1,2,2-tetrachloroethane	Industrial solvents
1,2-dibromoethane	Pesticides and fumigants
1,2-Dichloroethane	Industrial solvents
1,2-Dichlorotetrafluoroethane	Industrial solvents
1,2,4-trichlorobenzene	Industrial solvents
1,2,4-trimethylbenzene	Industrial solvents
1,3-Butadiene	Coatings and plastics
1,3-dichlorobenzene	Pesticides and fumigants
1,3,5-trimethylbenzene	Industrial solvents
1,4-dichlorobenzene	Pesticides and fumigants
1,4-Dioxane	Industrial solvents
2-(2-ethoxyethoxy)-ethanol acetate	Industrial solvents
2-butenal	Industrial solvents
2-butoxy-ethanol	Plant metabolites
2-decenal	Plant metabolites
2-ethoxy-2-methyl-propane	Plant metabolites
2-ethyl-hexanoic acid	Plant metabolites
2-ethylhexanol	Plant metabolites
2-Furanmethanol	Plant metabolites
2-heptanone	Microbial metabolites
2-Hydroxy-3-pentanone	Unknown
2-iodo-ethanol	Unknown
2-methoxy-1-propanol	Coatings and plastics
2-methyl-1-propanol	Microbial metabolites
2-methyl-2-butanol	Plant metabolites
2-methyl-3-buten-2-ol (40%)	Plant metabolites
2-methyl-propanoic acid	Plant metabolites
2-methylbutanal	Microbial metabolites
2-methylbutane	Industrial solvents
2-methylbutanoic acid	Plant metabolites
2-methylbutanol	Microbial metabolites
2-methylfuran	Plant metabolites
2-pinen-4-one	Plant metabolites
2,2,4-trimethyl-1,3-pentanediol diisobutyrate	Coatings and plastics
2,3-Butanediol_isomer1	Microbial metabolites
2,3-Butanediol_isomer2	Microbial metabolites
2,3-Butanedione	Microbial metabolites
2,4-dimethyl-1-heptene	Animal metabolites
3-carene	Plant metabolites
3-hydroxy-2-butanone	Microbial metabolites
3-hydroxy-2,4,4-trimethylpentyl-2-methylpropanoate	Plant metabolites
3-methylbutanoic acid	Microbial metabolites
3-methylbutanol	Plant and microbial metabolites
4-Ethyltoluene	Plant metabolites

Continued on next page

Continued from previous page

Compound	Putative.source
4-isopropylcyclohex-2-en-1-one	Plant metabolites
4-isopropylcyclohexanol	Plant metabolites
4-Isopropylcyclohexanone	Unknown
4-methylhexanal	Unknown
5-ethyldihydro-2(3H)-furanone	Plant metabolites
Acetaldehyde	Plant and microbial metabolites
Acetic acid	Microbial metabolites
Acetone	Plant and microbial metabolites
acetophenone	Industrial solvents
Alpha-phellandrene	Plant metabolites
Alpha-pinene	Plant metabolites
alpha-terpinene	Plant metabolites
Alpha-thujene	Plant metabolites
Benzaldehyde	Industrial solvents
Benzene	Industrial solvents
Benzoic acid	Plant and microbial metabolites
Benzothiazole	Plant and microbial metabolites
beta-Myrcene	Plant metabolites
Beta-phellandrene	Plant metabolites
Beta-pinene	Plant metabolites
Bromodichloromethane	Industrial solvents
Bromoform	Industrial solvents
Bromomethane	Pesticides and fumigants
Butanal	Plant and microbial metabolites
Butanoic acid	Plant and microbial metabolites
Camphene	Plant metabolites
Caprolactam	Coatings and plastics
Carbon disulfide	Industrial solvents
Carbon Tetrachloride	Industrial solvents
Chlorobenzene	Industrial solvents
Chloroethane	Industrial solvents
Chloroform	Industrial solvents
Cis-1,2-Dichloroethene	Industrial solvents
Cis-1,3-dichloropropene	Pesticides and fumigants
Cyclohexane	Industrial solvents
Cyclopentane	Industrial solvents
Decanal	Plant metabolites
Dibromochloromethane	Plant metabolites
Dichlorodifluoromethane	Industrial solvents
Difluorodimethylsilane	Unknown
Dimethyl sulfide	Plant and microbial metabolites
Dimethyl sulfone	Plant metabolites
Dimethyl Sulfoxide (DMSO)	Industrial solvents
Dowanol 62b	Industrial solvents
Ethanol	Plant and microbial metabolites
Ethyl Acetate	Plant and microbial metabolites
Ethylbenzene	Industrial solvents
Fenchol	Plant metabolites
Fenchone	Plant metabolites
Formic acid	Pesticides and fumigants

Continued on next page

Continued from previous page

Compound	Putative.source
Furfural	Plant metabolites
Gamma-terpinene	Plant metabolites
Heptane	Plant metabolites
Hexachloro-1,3-Butadiene	Industrial solvents
hexamethyl-cyclotrisiloxane	Industrial solvents
Hexanal	Plant metabolites
Hexane	Industrial solvents
Hexanoic acid	Plant and microbial metabolites
Indole	Plant and microbial metabolites
Isoprene	Plant metabolites
Isopropanol	Industrial solvents
Limonene	Plant metabolites
m-,p-Xylene	Industrial solvents
Methanesulfonic acid anhydride (NI)	Microbial metabolites
Methyl acetate	Plant metabolites
Methyl butyl ketone	Plant metabolites
Methyl ethyl ketone	Industrial solvents
Methyl Isobutyl Ketone	Industrial solvents
Methyl methacrylate	Coatings and plastics
Methyl tert-butyl ether	Industrial solvents
Myrtenal	Plant metabolites
Naphthalene	Plant metabolites
NI_1	Unknown
NI_10	Unknown
NI_11	Unknown
NI_12	Unknown
NI_13	Unknown
NI_14	Unknown
NI_15	Unknown
NI_2	Unknown
NI_3	Unknown
NI_4	Unknown
NI_5	Unknown
NI_6 (terpenoid)	Plant metabolites
NI_7	Unknown
NI_8	Unknown
NI_9	Unknown
Nonanal	Plant metabolites
o-cymene	Plant metabolites
o-Xylene	Industrial solvents
Octanal	Plant and microbial metabolites
Octanoic acid	Microbial metabolites
Pentadecane	Plant metabolites
Pentanal	Plant metabolites
Pentanoic acid	Plant metabolites
Phellandral	Plant metabolites
Pinocarveol	Plant metabolites
Propanoic acid	Microbial metabolites
Propylene Glycol	Industrial solvents
Styrene	Coatings and plastics

Continued on next page

Continued from previous page

Compound	Putative.source
Terpinolene	Plant metabolites
Tetrachloroethylene	Coatings and plastics
Tetrahydrofuran	Industrial solvents
Toluene	Industrial solvents
Trans-1,2-dichloroethene	Industrial solvents
Trans-1,3-Dichloropropene	Pesticides and fumigants
tri(1,2-propyleneglycol), monomethyl ester	Unknown
Trichloroethylene	Industrial solvents
Trichlorofluoromethane	Industrial solvents
Trimethyl-silanol	Unknown
Trimethylsilyl fluoride	Unknown
Vinyl acetate	Coatings and plastics

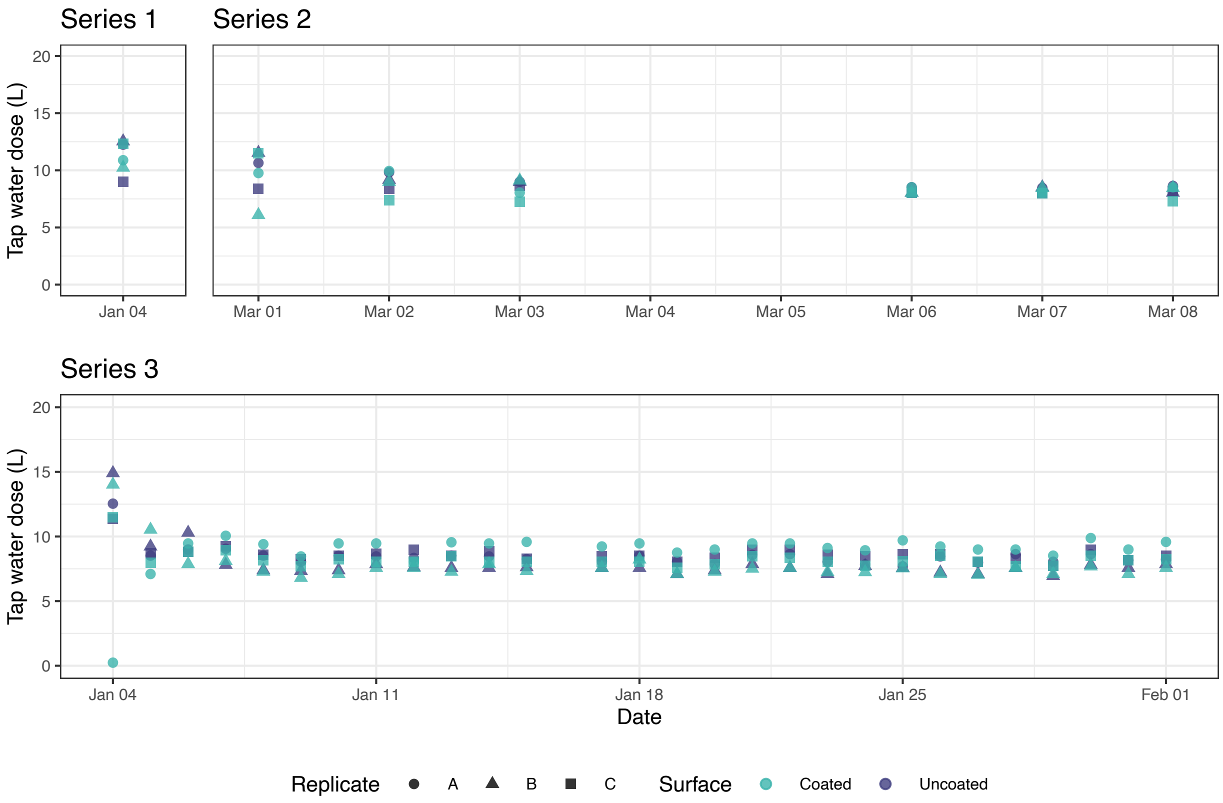

Figure S1. Dosages of tap water sprayed on the top surface of CLT blocks during wetting periods for each series.

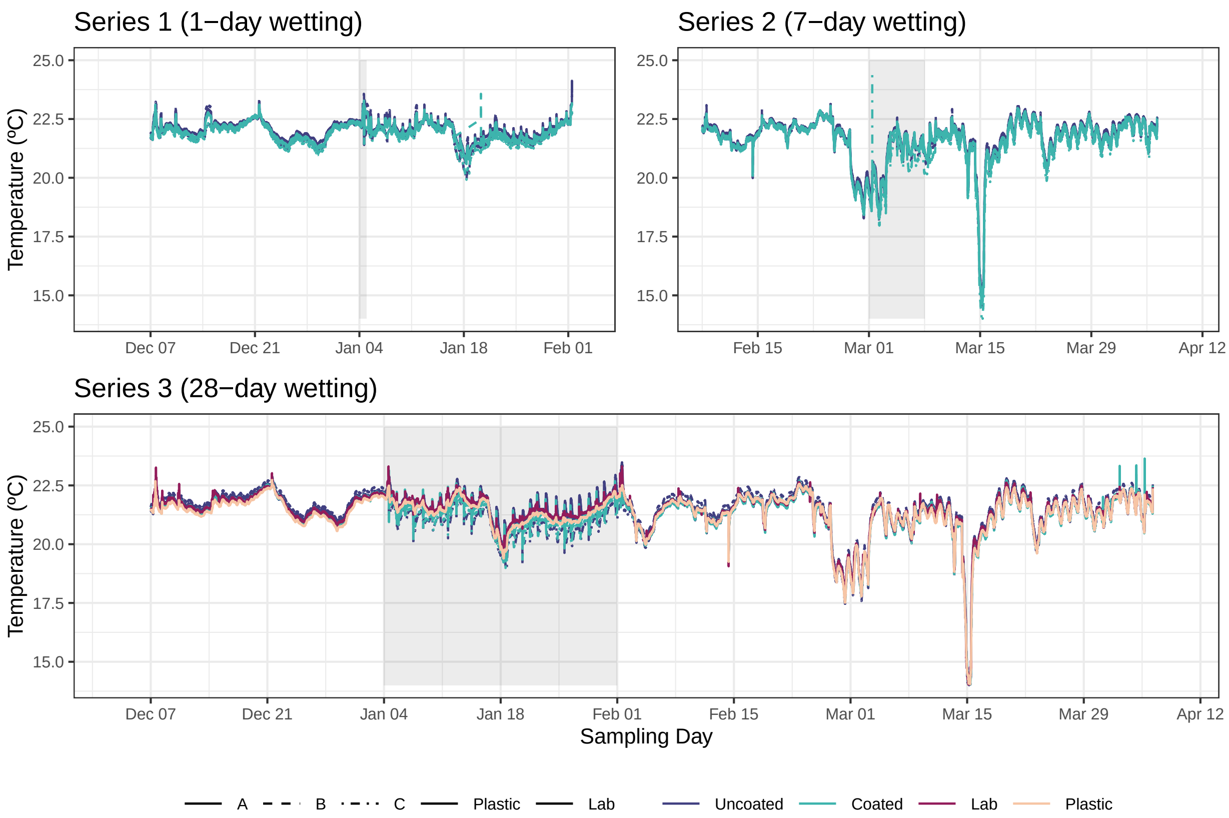

Figure S2. Temperatures inside microcosms and the external laboratory space for each series throughout the experiment. Shaded areas represent wetting periods.

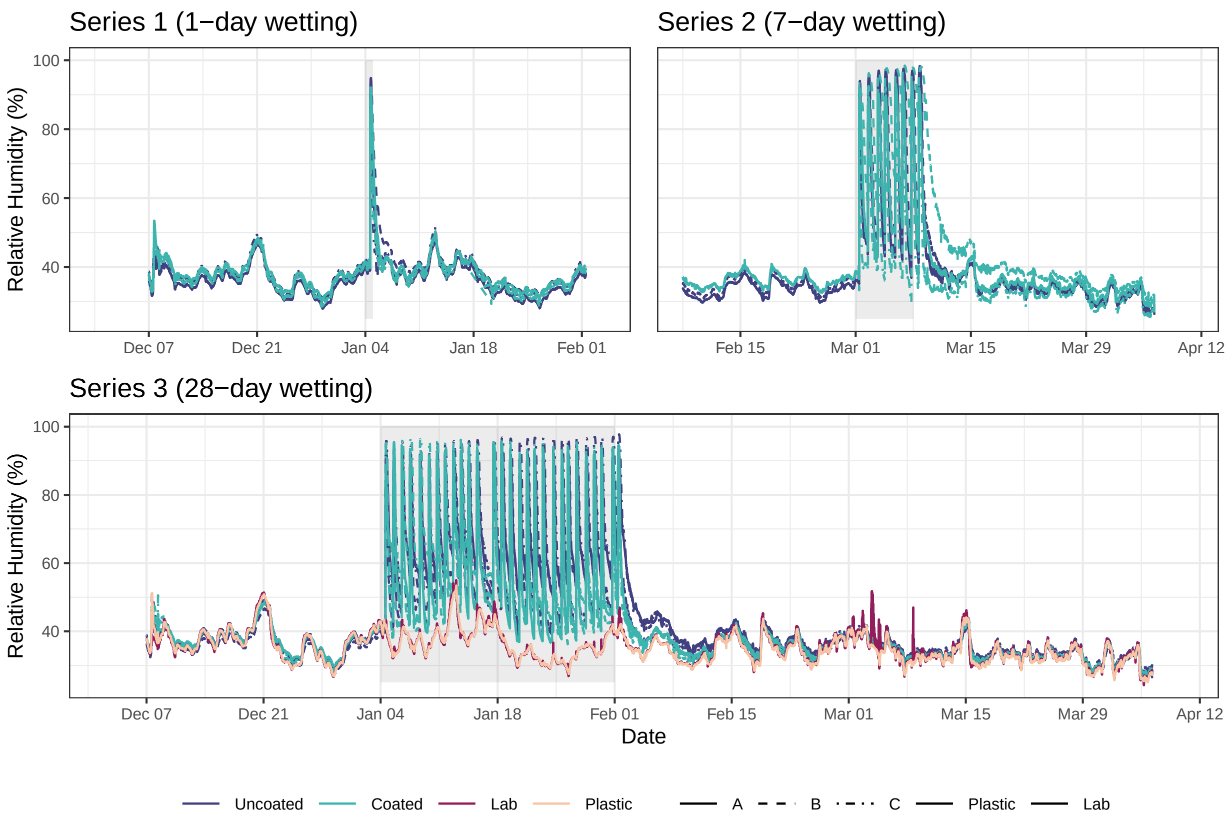

Figure S3. RH inside microcosms and the external laboratory space for each series throughout the experiment. Shaded areas represent wetting periods.

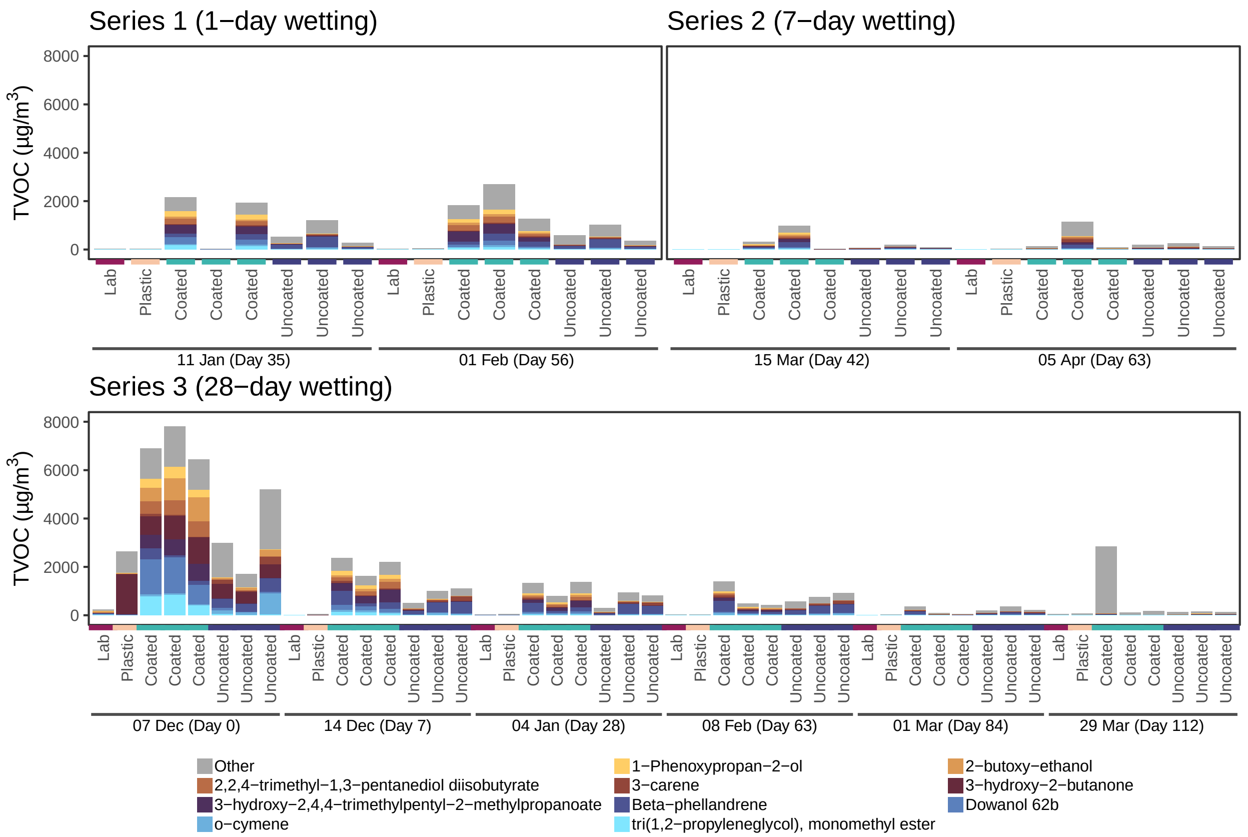

Figure S4. TVOC composition showing the top 10 most abundant VOCs; less abundant VOCs are lumped together within the category 'Other.' Bars represent individual samples. Within each series, bars are organized by sampling day, then by sample type. There are three replicates each day for coated and uncoated CLT blocks, and only a single sample each day for the plastic control microcosm and one for the external laboratory.

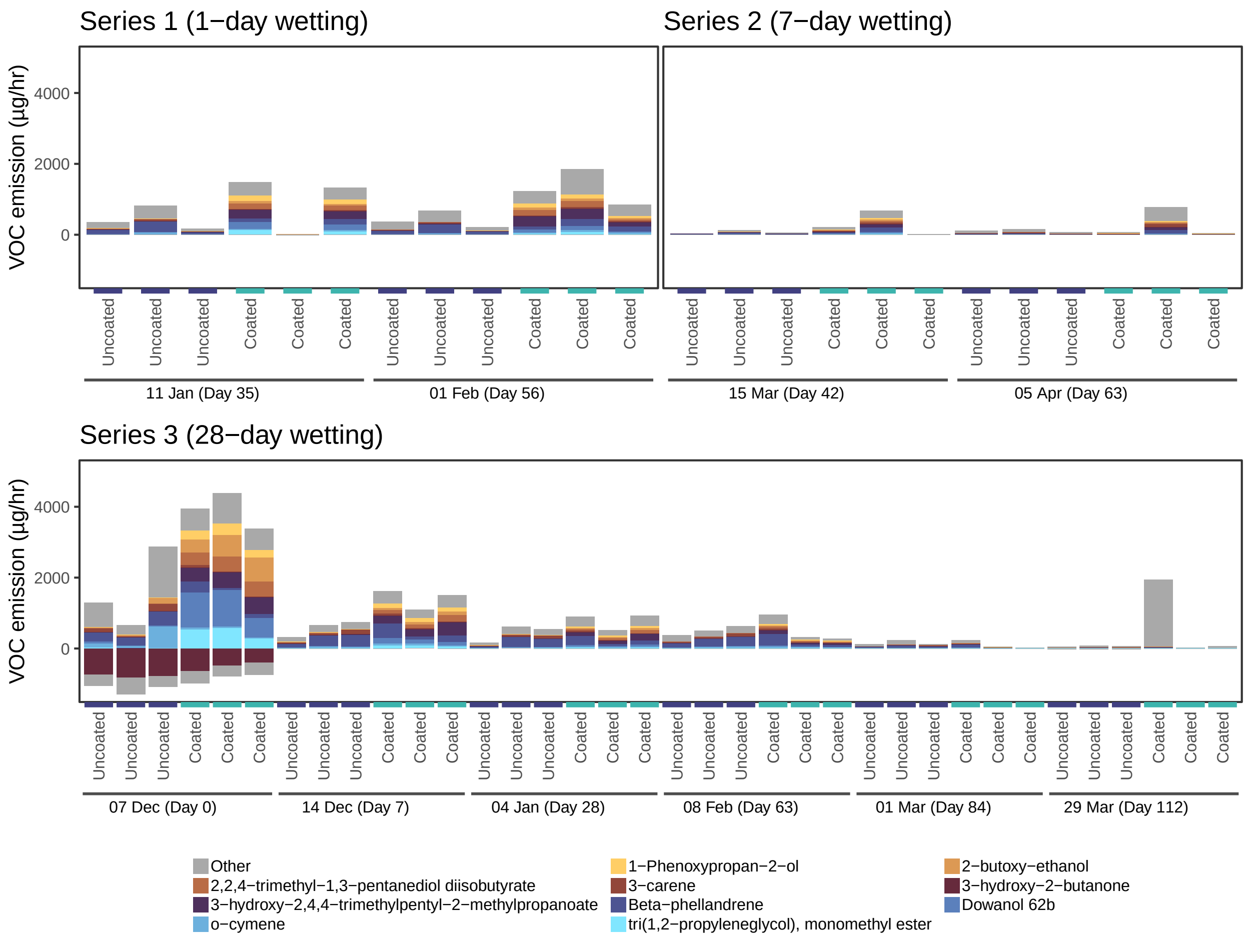

Figure S5. TVOC emission rates showing the top 10 most abundant VOCs; less abundant VOCs are lumped together within the category 'Other.' Bars represent individual samples. Within each series, bars are organized by sampling day, then by sample type. There are three replicates each day for coated and uncoated CLT blocks, and only a single sample each day for the plastic control microcosm and one for the external laboratory.

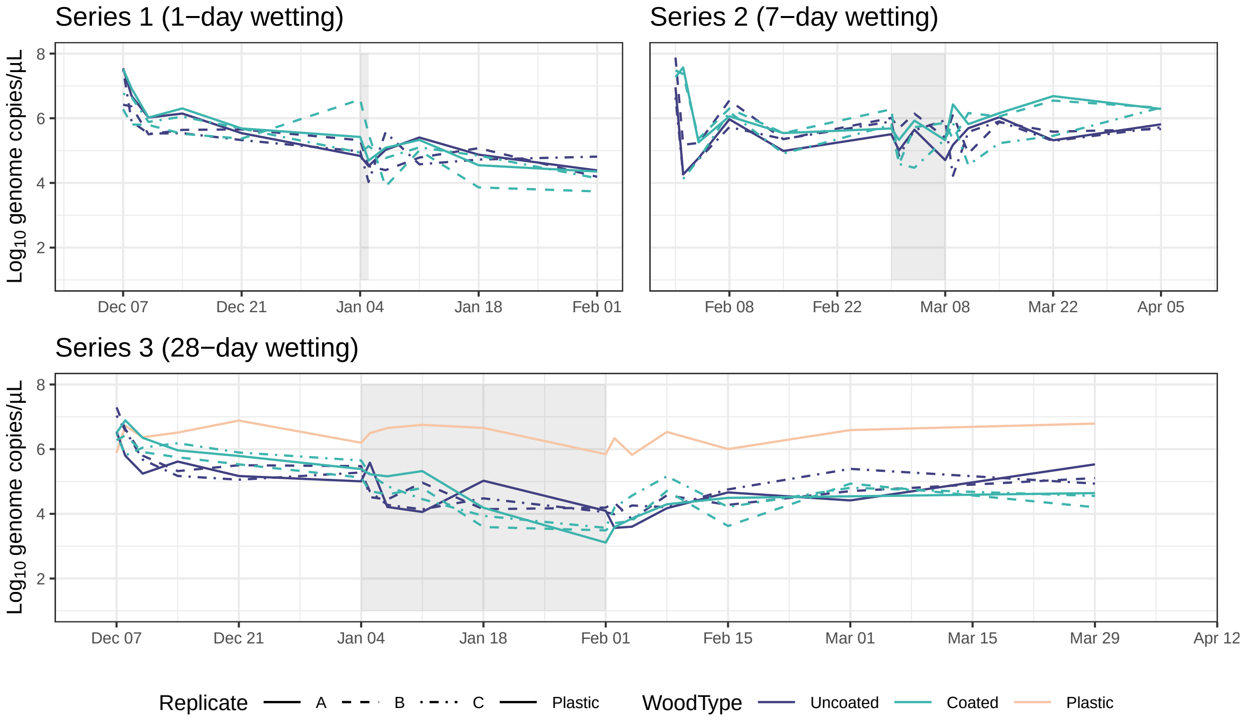

Figure S6. Total bacterial abundance (estimated by gene copy numbers) over time for each series. Data for the plastic control box shown on all three series as a reference. Shaded areas represent wetting periods.

Table S2. Summary statistics of viable to total bacterial abundance ratios for each series and surface type.

Series	WoodType	mean	min	max	sd
1	Uncoated	0.016	0.000221	0.32	0.055
1	Coated	0.018	0.000032	0.15	0.035
2	Uncoated	0.053	0.000100	0.51	0.120
2	Coated	0.058	0.000110	0.74	0.170
3	Uncoated	0.049	0.000108	0.31	0.083
3	Coated	0.032	0.000024	0.2	0.041
3	Plastic	0.017	0.000280	0.12	0.030

Table S3. Alpha diversity measures (Observed, Shannon index, inverse Simpson) and standard error for archaea by surface type and sampling period (before, during, after wetting).

WoodType	Period	count	Obs_mean	Obs_se	Shan_mean	Shan_se	Simp_mean	Simp_se
H2O	NA	4	236	39	4.5	0.29	0.983	0.004
Uncoated	Before	14	142	13	3.7	0.12	0.951	0.008
Uncoated	During	11	178	13	3.94	0.1	0.97	0.003
Uncoated	After	14	205	13	4.23	0.1	0.976	0.003
Coated	Before	15	122	14	3.42	0.08	0.936	0.008
Coated	During	12	181	16	4.06	0.13	0.971	0.004
Coated	After	15	156	9	3.78	0.13	0.958	0.008
Plastic	NA	13	170	16	3.95	0.19	0.958	0.01

Table S4. Alpha diversity measures (Observed, Shannon index, inverse Simpson) and standard error for bacteria by surface type and sampling period (before, during, after wetting).

WoodType	Period	count	Obs_mean	Obs_se	Shan_mean	Shan_se	Simp_mean	Simp_se
H2O	NA	4	5901	520	4.67	0.16	0.892	0.016
Uncoated	Before	14	4726	251	2.55	0.37	0.758	0.037
Uncoated	During	11	5348	174	3.96	0.43	0.873	0.035
Uncoated	After	14	5753	168	3.26	0.43	0.816	0.038
Coated	Before	15	4313	291	2.08	0.25	0.737	0.028
Coated	During	12	5349	232	3.97	0.32	0.878	0.025
Coated	After	15	4999	156	3.24	0.31	0.821	0.023
Plastic	NA	13	5177	271	3.18	0.39	0.825	0.031

Table S5. Alpha diversity measures (Observed, Shannon index, inverse Simpson) and standard error for fungi by surface type and sampling period (before, during, after wetting).

WoodType	Period	count	Obs_mean	Obs_se	Shan_mean	Shan_se	Simp_mean	Simp_se
H2O	NA	4	74	4	2.35	0.19	0.732	0.064
Uncoated	Before	14	66	2	2.79	0.11	0.843	0.028
Uncoated	During	11	71	1	2.83	0.12	0.861	0.024
Uncoated	After	14	73	0.8	3.0	0.12	0.864	0.028
Coated	Before	15	61	2	2.88	0.1	0.875	0.024
Coated	During	12	70	1	2.94	0.09	0.866	0.017
Coated	After	15	68	1	2.61	0.13	0.802	0.039
Plastic	NA	13	71	2	2.58	0.11	0.775	0.027

Table S6. Alpha diversity measures (Observed, Shannon index, inverse Simpson) and standard error for virus by surface type and sampling period (before, during, after wetting).

WoodType	Period	count	Obs_mean	Obs_se	Shan_mean	Shan_se	Simp_mean	Simp_se
H2O	NA	4	502	208	2.68	0.28	0.775	0.091
Uncoated	Before	14	138	23	2.45	0.18	0.753	0.037
Uncoated	During	11	195	28	2.7	0.17	0.805	0.038
Uncoated	After	14	275	43	2.23	0.24	0.671	0.045
Coated	Before	15	107	20	2.52	0.09	0.815	0.021
Coated	During	12	204	35	2.43	0.17	0.74	0.042
Coated	After	15	150	18	2.29	0.1	0.75	0.028
Plastic	NA	13	208	42	2.14	0.19	0.677	0.039

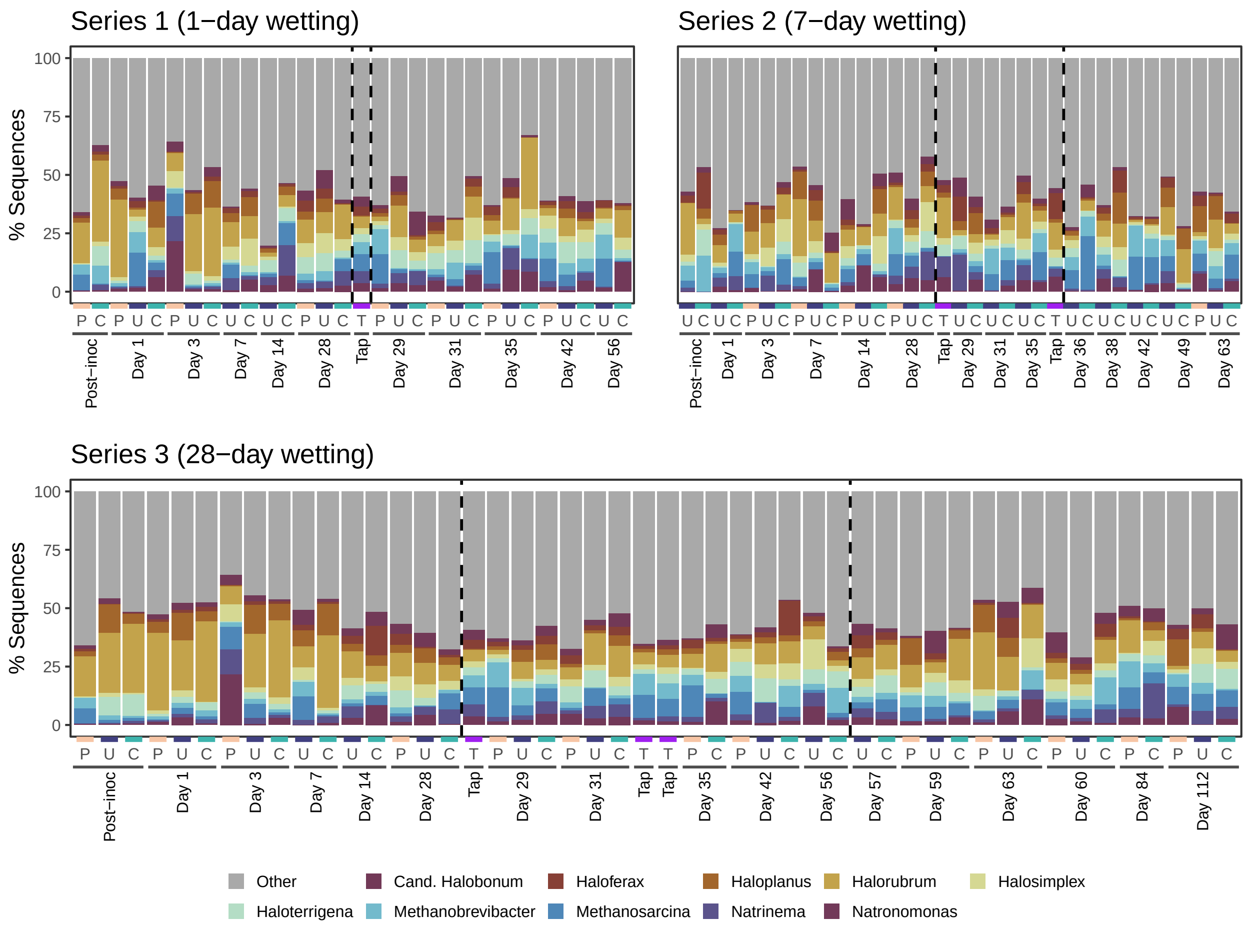

Figure S7. Relative abundance of the top 10 most abundant archaeal genera identified in each sample for each series. All other genera were aggregated into category "Other." Samples are organized first by sampling day, then by sample type (P = Plastic, U = Uncoated, C = Coated, T = Tap water). Wetting period start and end days are indicated by dashed vertical lines. Tap water and control samples are shown in each series where relevant for reference.

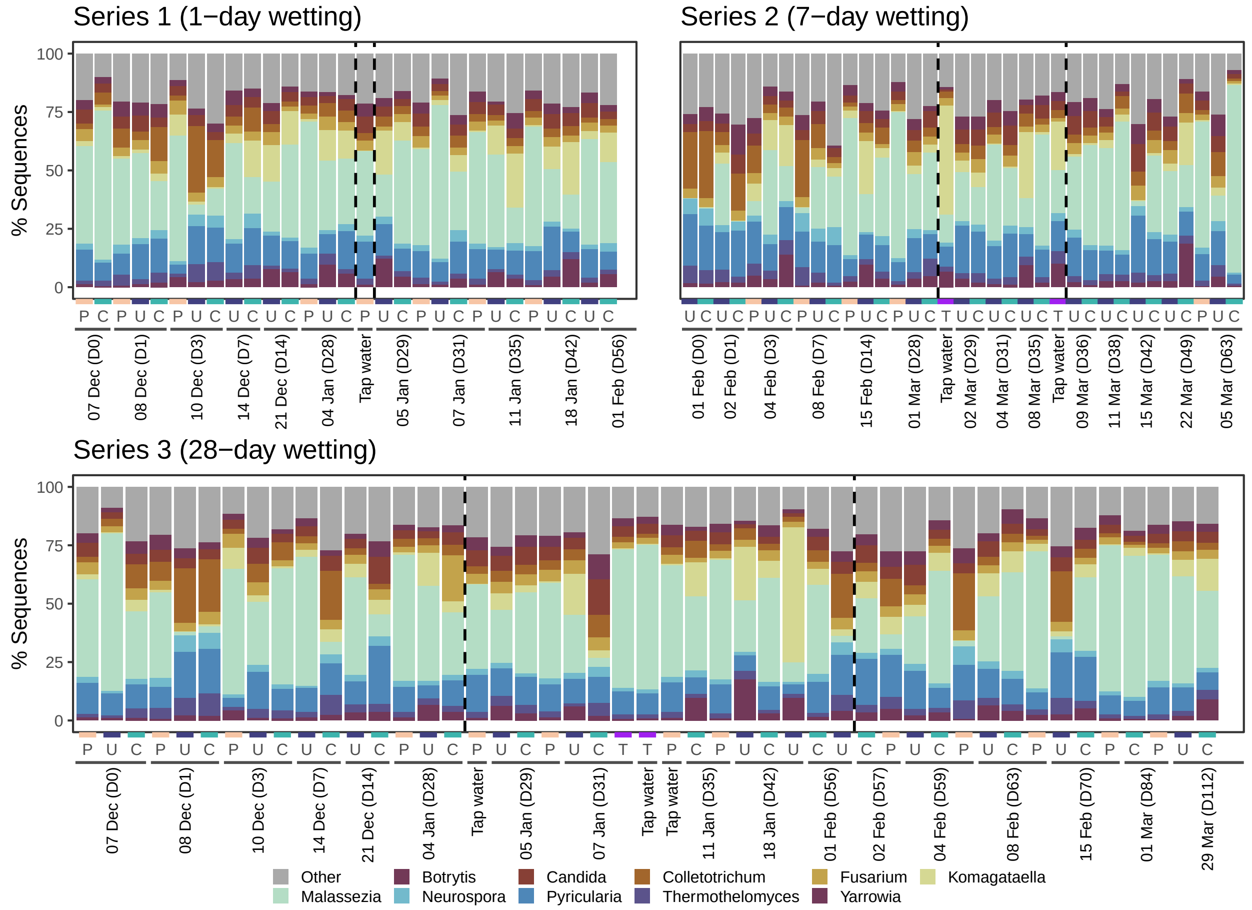

Figure S8. Relative abundance of the top 10 most abundant fungal genera identified in each sample for each series. All other genera were aggregated into category “Other.” Samples are organized first by sampling day, then by sample type (P = Plastic, U = Uncoated, C = Coated, T = Tap water). Wetting period start and end days are indicated by dashed vertical lines. Tap water and control samples are shown in each series where relevant for reference.

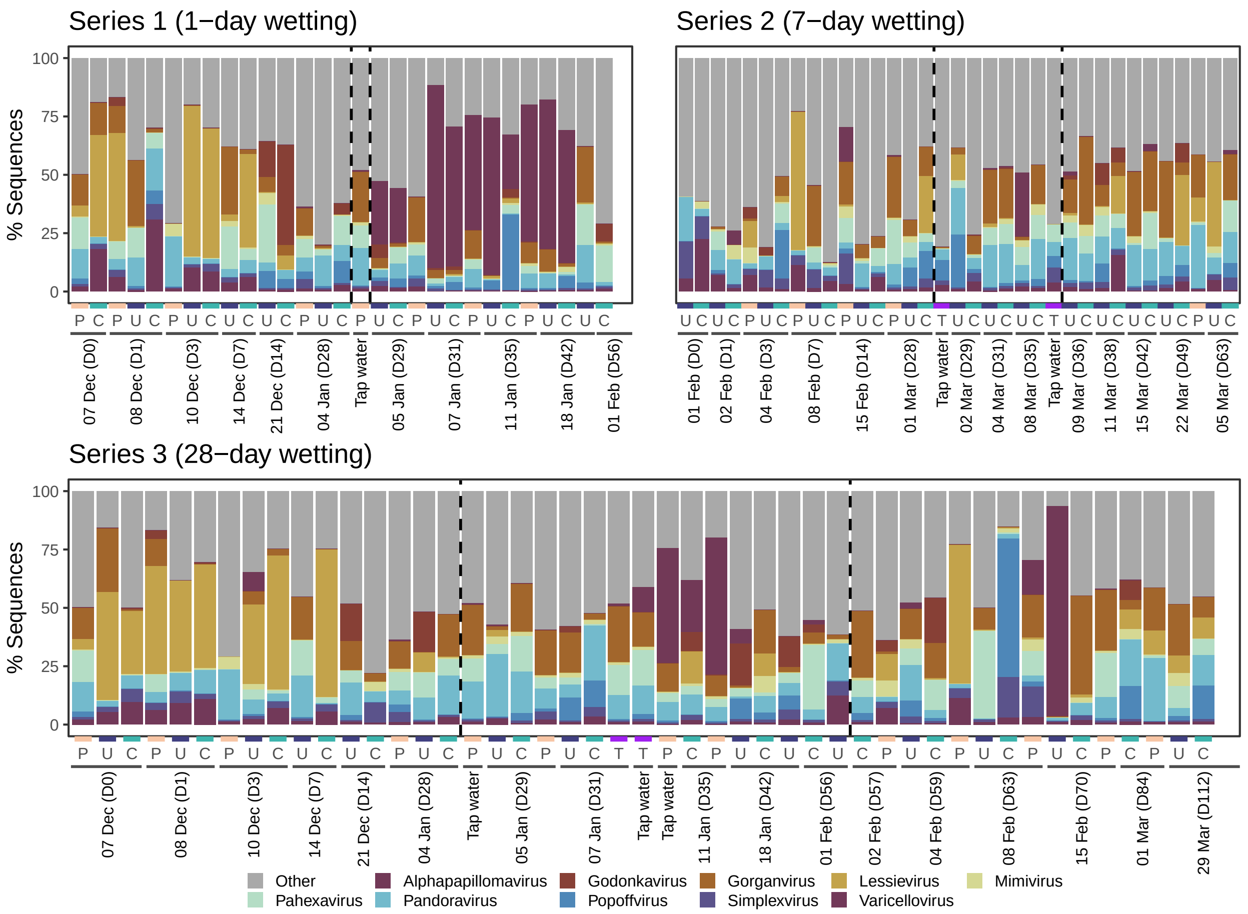

Figure S9. Relative abundance of the top 10 most abundant viral genera identified in each sample for each series. All other genera were aggregated into category "Other." Samples are organized first by sampling day, then by sample type (P = Plastic, U = Uncoated, C = Coated, T = Tap water). Wetting period start and end days are indicated by dashed vertical lines. Tap water and control samples are shown in each series where relevant for reference.

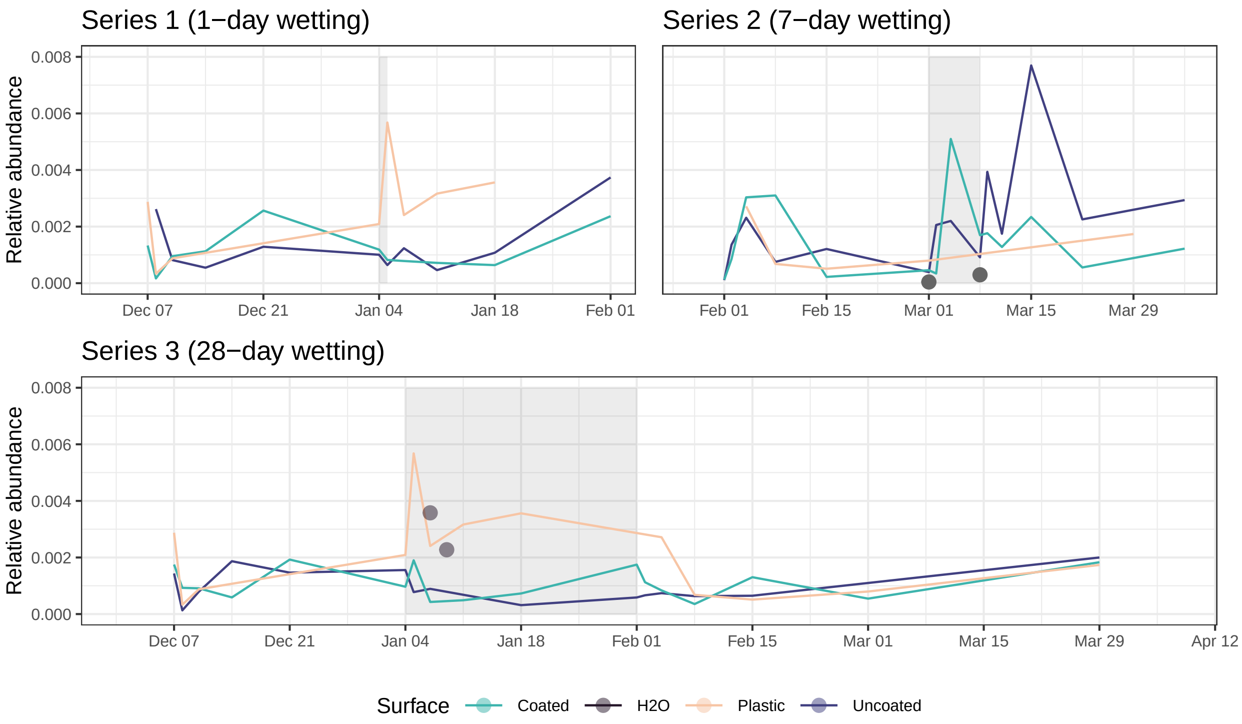

Figure S10. Relative abundance of *E. coli* in samples from coated and uncoated CLT blocks, the plastic control microcosm, and tap water (grey circles). Shaded areas represent wetting periods. Note that there was only a single plastic control microcosm; its data are shown on all three series for reference.

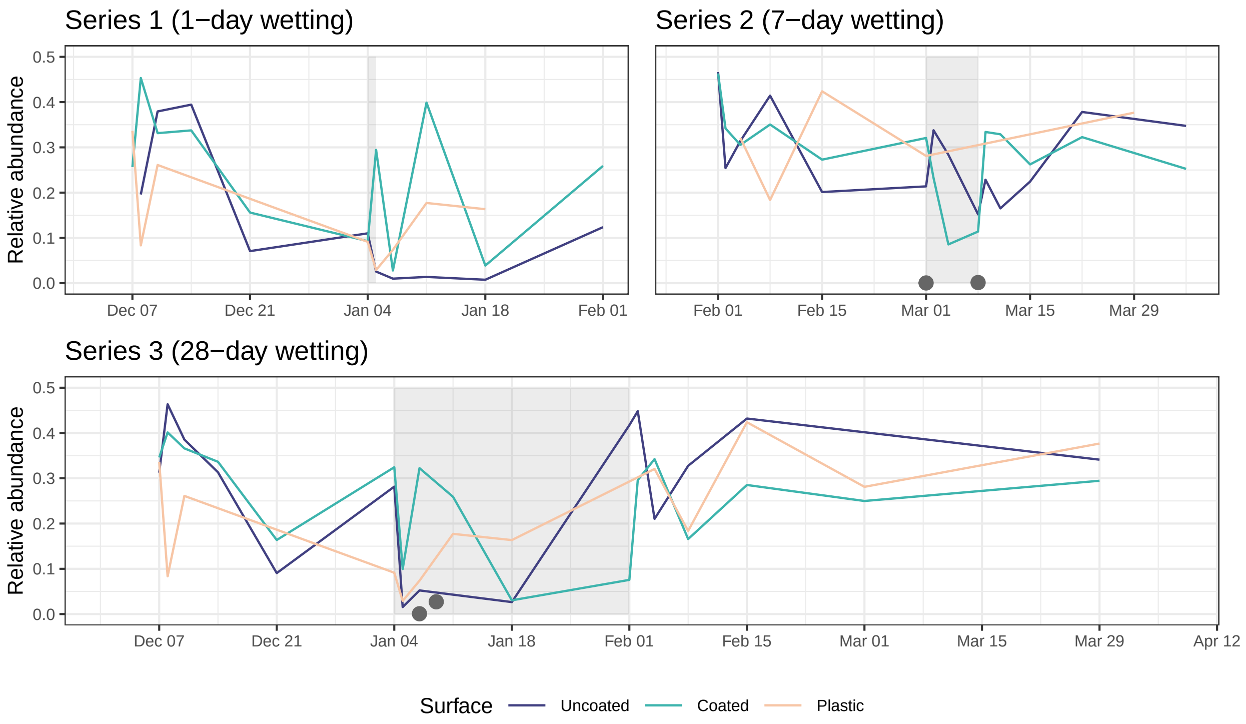

Figure S11. Relative abundance of *P. fluorescens* in samples from coated and uncoated CLT blocks, the plastic control microcosm, and tap water (grey circles). Shaded areas represent wetting periods. Note that there was only a single plastic control microcosm; its data are shown on all three series for reference.

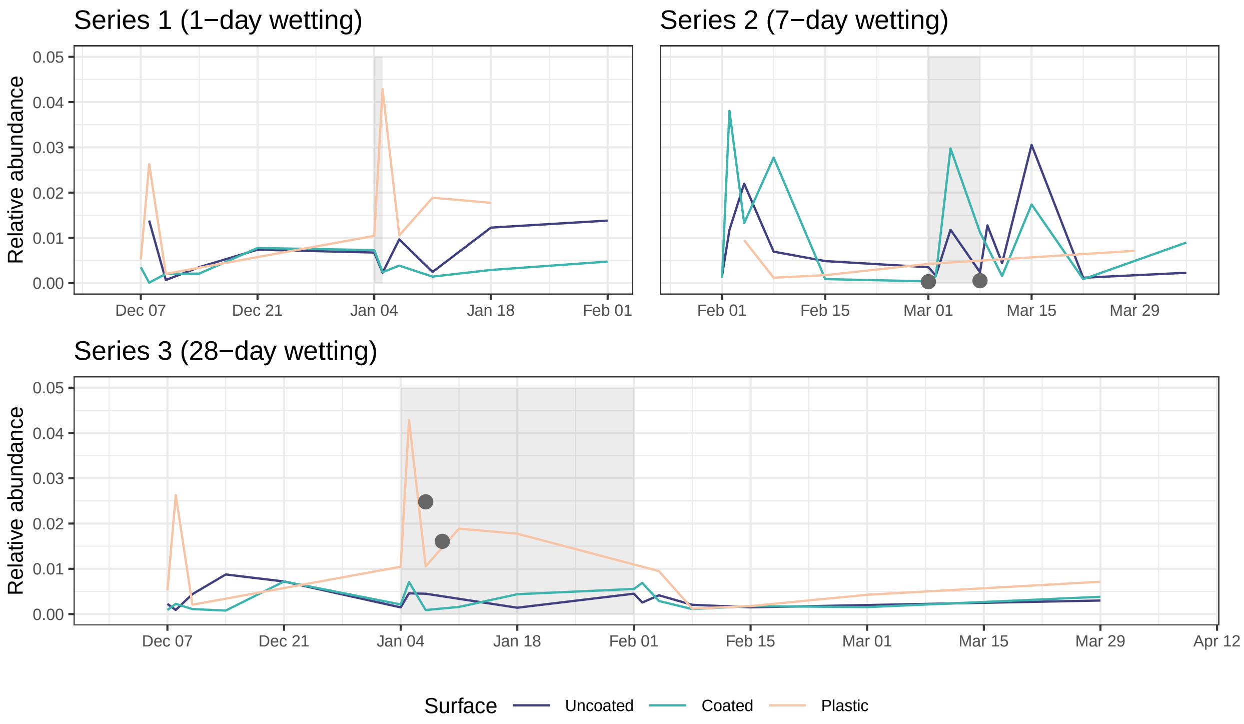

Figure S12. Relative abundance of *S. aureus* in samples from coated and uncoated CLT blocks, the plastic control microcosm, and tap water (grey circles). Shaded areas represent wetting periods. Note that there was only a single plastic control microcosm; its data are shown on all three series for reference.

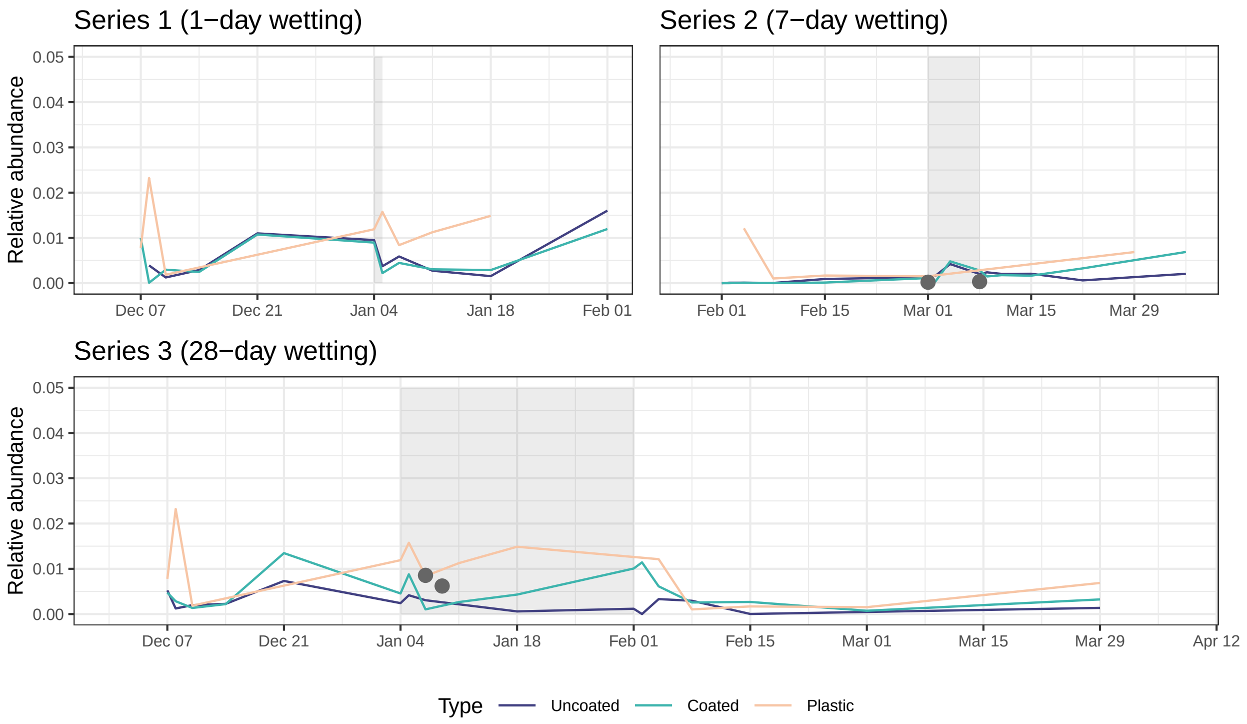

Figure S13. Relative abundance of *S. epidermis* in samples from coated and uncoated CLT blocks, the plastic control microcosm, and tap water (grey circles). Shaded areas represent wetting periods. Note that there was only a single plastic control microcosm; its data are shown on all three series for reference.
